# GPU accelerated adaptive banded event alignment for rapid comparative nanopore signal analysis

**DOI:** 10.1186/s12859-020-03697-x

**Published:** 2020-08-05

**Authors:** Hasindu Gamaarachchi, Chun Wai Lam, Gihan Jayatilaka, Hiruna Samarakoon, Jared T. Simpson, Martin A. Smith, Sri Parameswaran

**Affiliations:** 1 School of Computer Science and Engineering, UNSW Sydney, Sydney, Australia; 2 Kinghorn Centre for Clinical Genomics, Garvan Institute of Medical Research, Sydney, Australia; 3 Department of Computer Engineering, University of Peradeniya, Peradeniya, Sri Lanka; 4 Ontario Institute for Cancer Research, Toronto, Canada; 5 Department of Computer Science, University of Toronto, Toronto, Canada; 6 St-Vincent’s Clinical School, Faculty of Medicine, UNSW Sydney, Sydney, Australia; 7 CHU Sainte-Justine Research Centre, Montreal, Canada; 8 Department of Biochemistry and Molecular Medicine, Faculty of Medicine, University of Montreal, Montreal, Canada

**Keywords:** Nanopore, Signal alignment, Event alignment, Methylation, GPU, GPU acceleration, Optimisation, SoC, Nanopolish, f5c

## Abstract

**Background:**

Nanopore sequencing enables portable, real-time sequencing applications, including point-of-care diagnostics and in-the-field genotyping. Achieving these outcomes requires efficient bioinformatic algorithms for the analysis of raw nanopore signal data. However, comparing raw nanopore signals to a biological reference sequence is a computationally complex task. The dynamic programming algorithm called Adaptive Banded Event Alignment (ABEA) is a crucial step in polishing sequencing data and identifying non-standard nucleotides, such as measuring DNA methylation. Here, we parallelise and optimise an implementation of the ABEA algorithm (termed *f5c*) to efficiently run on heterogeneous CPU-GPU architectures.

**Results:**

By optimising memory, computations and load balancing between CPU and GPU, we demonstrate how *f5c* can perform ∼3-5 × faster than an optimised version of the original CPU-only implementation of ABEA in the *Nanopolish* software package. We also show that *f5c* enables DNA methylation detection on-the-fly using an embedded System on Chip (SoC) equipped with GPUs.

**Conclusions:**

Our work not only demonstrates that complex genomics analyses can be performed on lightweight computing systems, but also benefits High-Performance Computing (HPC). The associated source code for *f5c* along with GPU optimised ABEA is available at https://github.com/hasindu2008/f5c.

## Background

Advances in genomic technologies have improved the feasibility and accessibility of rapid species identification, accurate clinical diagnostics, and specialised therapeutics, amongst other applications. The latest generation (third generation) of sequencing technologies generate data in the order of terabytes. Oxford Nanopore Technologies’ (ONT) pocket-sized MinION device generates ∼1 TB of raw signal data during a typical sequencing run, while their high-throughput PromethION device can generate >50TB of data in <60h. Computational analysis of such massive data currently poses a challenge.

**Fig. 1:**
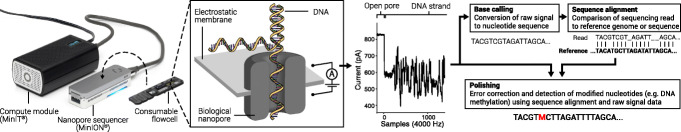
Nanopore DNA sequencing and associated data analysis. A consumable flowcell containing an array of hundreds or thousands of such nanopores is loaded into the sequencing device (e.g. MinION). Ionic current (in pico amperes) is measured when DNA strands pass through nanopores to produce the *raw signal*, which is eventually basecalled. The base-called reads are then aligned to a reference genome. The raw signal is then revisited during the polishing step. Images of nanopore devices are reproduced with permission from ONT

Nanopore sequencing measures characteristic disruptions in the electric current (referred to hereafter as *raw signal*) when DNA passes through a nanopore (Fig. 1). The instantaneous current measured in the R9.4.1 pore model depends on 5-6 contiguous bases [1]. The measured signal also presents stochastic noise due to a number of factors [2]. Additionally, the speed of the DNA strand moving through the pore can vary, causing the signal to warp in the time domain [2]. The *raw signal* is converted to nucleotide strings (reads) through a process called base-calling (Fig. 1). Despite recent improvements, nanopore base-calling often introduces errors (∼3-5% at the time of writing) given the use of probabilistic methods to infer biological sequences from often noisy raw signal [3]. To overcome base-calling errors, raw signal can be revisited to improve the reconstitution of the base-called sequence *a posteriori* (Fig. 1). This process, termed ‘polishing’, can correct base-calling errors by aligning the raw signal to a biological reference sequence [4, 5], thus identifying idiosyncrasies in the raw signal by comparing observed signal levels to the expected levels at all aligned positions. Polishing can also reveal base substitutions (i.e. mutations) or base modifications such as 5-methylcytosine (5mC), a dynamic biochemical modification of DNA that is associated with genetic activity and regulation [6]. Detecting 5mC bases is important for the study of DNA methylation in the field of epigenetics [7].

A crucial algorithmic component of polishing is the alignment of raw signal—a time series of electric current—to a biological reference sequence. One of the first and most popular raw nanopore signal alignment algorithms is implemented in *Nanopolish* [6], which employs a dynamic programming strategy referred to as Adaptive Banded Event Alignment (ABEA). ABEA is one of the most time consuming steps during the process of analysing raw nanopore data. For instance, in-house profiling revealed that when performing methylation detection with *Nanopolish*, the ABEA step alone consumes ∼70% of the total CPU time. Considering the increasing amount of data generated by high-throughput nanopore sequencers, solutions are required to accelerate ABEA and reduce the turnaround time of certain nanopore sequencing applications, such as real-time polishing or methylation detection.

In this study, we describe and dissect the ABEA algorithm in detail to optimise and parallelise its execution to exploit heterogeneous CPU-GPU architectures, commonplace in mainstream computing systems. We demonstrate the utility of our GPU-optimised ABEA by incorporating a completely re-engineered version of the popular methylation detection tool *Nanopolish*. First, we modified the original *Nanopolish* methylation detection tool to efficiently utilise existing CPU resources, which we refer to as *f5c*. Then, we incorporated a GPU-optimised ABEA algorithm into *f5c*. We demonstrate how *f5c* enables DNA methylation detection using nanopore sequencers in real-time (i.e. on-the-fly processing of the output) by using a lightweight embedded computer system equipped with a GPU (e.g., NVIDIA Jetson TX2). We also demonstrate how *f5c* benefits a wide range of computing devices, from embedded systems and laptops to workstations and high performance servers. *f5c* is available at https://github.com/hasindu2008/f5c.

## The ABEA algorithm

**Fig. 2:**
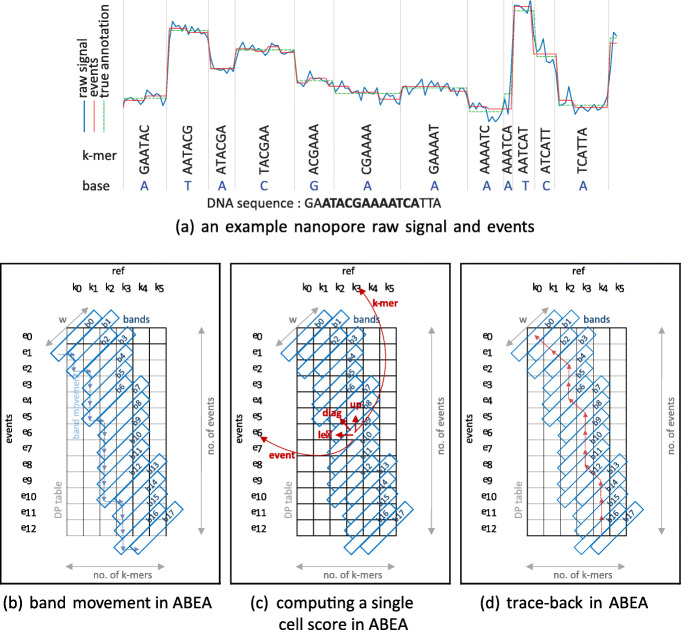
Nanopore raw signal, events and ABEA algorithm. In **a***events* are the result of the event detection step (time series segmentation of the raw signal based on abrupt changes — detailed in Supplementary Materials) and *true annotation* is the expected output of ABEA. In **b**, **c** and **d**, vertical axis represents the events and horizontal axis represents the *ref* k-mers (k-mers within the base-called read). The dynamic programming table (DP table) is for 13 events, indexed from *e*_0_−*e*_12_ vertically, and the *ref* k-mers, indexed from *k*_0_−*k*_5_ horizontally. For computational and memory efficiency, only the diagonal bands (marked using blue rectangles) with a band width of *W* (typically *W*=100 for nanopore signals) are computed. The bands are computed along the diagonal from top-left (*b0*) to bottom-right (*b17*). Each cell score is computed in function of five factors: scores from the three neighbouring cells (up, left and diagonal); the corresponding *ref* k-mer; and, the event (shown for the cell *e*_6_, *k*_3_ via red arrows in c). Observe that all the cells in the *n*^th^ band can be computed in parallel as long as the *n*−1^th^ and *n*−2^th^ bands are computed beforehand. To contain the optimal alignment, the band adapts by moving down or to the right as shown using blue arrows. The adaptive band movement is determined by the Suzuki-Kasahara heuristic rule [9]


ABEA was first introduced in the raw nanopore signal analysis package *Nanopolish* [6]. The origin of the ABEA algorithm can be tracked to the Smith-Waterman (SW) dynamic programming sequence alignment algorithm that was first described in 1981. The original SW algorithm has a computational complexity of *O*(*n*^2^) and is most practical when the sequences are very short. Several optimisations to SW have since been introduced. Heuristic approaches, such as banded SW, attempt to reduce the search space by limiting computation along the diagonal of the dynamic programming table [8]. While the banded approach is suitable for fast alignment of second-generation sequencing data—which are composed of relatively short reads—it is less so for third generation long reads, as significantly longer width is required to contain the alignment within the band. The more recent Suzuki-Kasahara (SK) algorithm [9] uses a heuristic optimisation to banded SW that allows the band to adapt and move during the alignment, thus containing the optimal alignment within the band while allowing large gaps in the alignment. The SK algorithm is well-suited for aligning long and error-prone third generation reads in base-space (nucleotide sequences). The SK alignment algorithm was later modified and extended to ABEA in *Nanopolish* to enable signal-space alignment of time series signal data instead of nucleotide sequences. A simplified example of the ABEA algorithm and a representative dynamic programming table is shown in Fig. 2. Algorithm 1 summarises the ABEA algorithm and the reader may refer to Supplementary Materials for a detailed explanation.

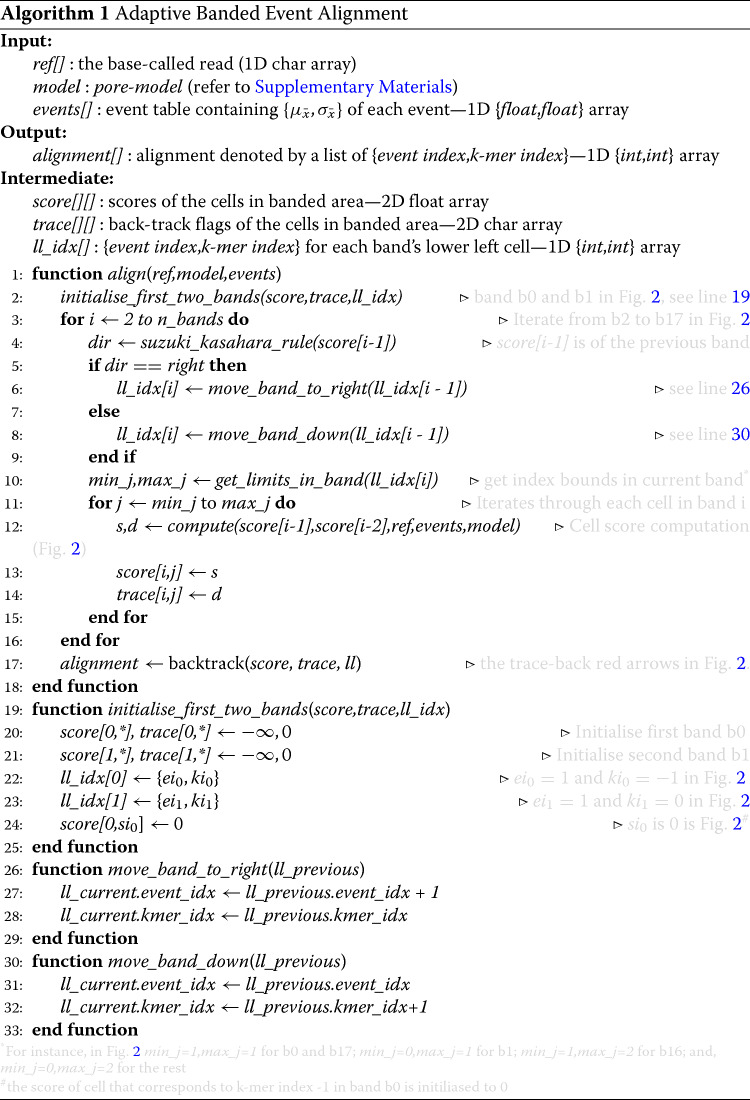


## Methods

### CPU-GPU optimisations

*f5c* employs a fork-join multi-threading model (with work stealing) implemented using C POSIX threads.

Implementing the ABEA algorithm for GPU execution is not a straightforward task due to three main factors: (i) inefficient memory access patterns, which are not ideal for GPUs with relatively less powerful and smaller caches (compared to CPUs), resulting in frequent instruction stalls; (ii) read lengths of the input vary significantly (from ∼100 bases to >1M bases), requiring millions to billions of dynamic memory allocations—an expensive operation in GPUs; and (iii) non uniform distribution of read lengths in the input causes irregular utilisation of GPU cores. These challenges were overcome by: (i) tailoring the algorithm and GPU user-managed cache to exploit cache friendly memory access patterns; (ii) employing a custom heuristic-based memory allocation scheme; and (iii) using a heuristic-based work partitioning and load balancing scheme between CPU and GPU.

The GPU implementation of ABEA algorithm was performed using CUDA C. A brief summary of our optimisations is listed below.

**Parallelisation and computational optimisations:** To achieve fast performance on GPUs, their thousands of tiny computing cores must be sufficiently occupied. For this, thousands of parallel threads must be launched, which requires thousands of parallel tasks. This is achieved by processing a batch of reads in parallel and concurrently computing all the cells of a dynamic programming matrix band (Fig. 2b, lines ??-?? in Algorithm 1). As the bandwidth is 100 cells, a read batch of a few hundred can sufficiently occupy thousands of GPU cores. GPU core utilisation is further enhanced by improving memory access latency by using the GPU’s fast cache memory (shared memory) and a technique called memory coalescing. The current, previous and 2^nd^ previous bands, which are frequently accessed by hundreds of threads in parallel, are kept in the shared memory. Data arrays such as the *score* array, *trace* array, reference k-mers and events that are in slow DRAM (global memory) are placed (laid out) such that contiguous threads access contiguous memory locations. This facilitates memory coalescing (one memory access can fetch data required by a large number of threads), consequently reducing the number of accesses to DRAM.

**Memory optimisation:** Dynamic memory allocations in the GPU memory are expensive and must be minimised for fast performance. We significantly reduced the number of dynamic memory allocations by employing a lightweight heuristic-based custom memory allocation scheme. In brief, large chunks of contiguous memory are pre-allocated when initiating the program to accommodate a batch of reads, which are then reused throughout the execution of the program. The sizes of these large chunks are determined by the available GPU memory and a heuristically determined value for the average number of events per base (i.e. average value of the number of events divided by the read length).

**Heterogeneous processing:** If all queried reads were of similar length, GPU threads that process the reads would complete approximately at the same time, and thus GPU cores will be equally busy throughout the execution. However, nanopore read length distributions can include reads which are significantly longer than the average read length. When the GPU threads process reads in parallel, longer reads cause all other GPU threads to wait until processing of the longest read is completed. These waiting threads lead to underutilisation of GPU cores. This issue is remedied by employing heterogeneous processing, where the CPU processes these very long reads while the GPU is processing the rest of the reads in parallel. CPU cores have a higher clock frequency than the GPU cores, therefore such very long reads can be independently and quickly processed by the CPU while the remaining reads are processed by GPU cores in parallel.

A detailed breakdown of these optimisations, experimental evidence that justify design and optimisation decisions—including a section describing the fundamentals of GPU architecture and programming—can be found in Supplementary Materials.

### Biological data analysis

Comparative performance benchmarking was performed using the publicly available NA12878 (human genome) “Nanopore WGS Consortium” sequencing data [4]. The datasets used for the experiments, their statistics (number of reads, total bases, mean read length and maximum read length) and their source are listed in Table 1. *D*_*small*_, a small subset, was used for testing a wide range of systems (all systems in Table 2, i.e. embedded system, low-end and high-end laptops, workstation and a high-performance server). Two complete nanopore MinION data sets (*D*_*ligation*_ and *D*_*rapid*_) are only tested on three systems due to the larger run-time and incidental access to the other two systems. *D*_*ligation*_ and *D*_*rapid*_ represent the two existing nanopore sample preparation methods (ligation and rapid [10]) that affects the read length distribution.

**Table 1:** Information of the datasets

**Dataset**	**Number of reads**	**Number of bases (Gbases)**	**Mean read length (Kbases)**	**Max read length (Kbases)**	**Source / SRA accession**
*D*_*small*_	19275	0.15	7.7	196	[11]
*D*_*ligation*_	451020	3.62	8.0	1500	ERR2184733
*D*_*rapid*_	270189	2.73	10.0	386	ERR2184734

**Table 2:** Different systems used for experiments

**System Name**	**Info**	**CPU**	**CPU cores/ threads**	**RAM (GB)**	**GPU**	**GPU mem (GB)**	**GPU arch**
SoC	NVIDIA Jetson TX2 embedded module	ARMv8 Cortex-A57 + NVIDIA Denver2	6 / 6	8	Tegra	shared with RAM	Pascal / 6.2
lapL	Acer F5-573G laptop	i7-7500U	2/4	8	Geforce 940M	4	Maxwell / 5.0
lapH	Dell XPS 15 laptop	i7-8750H	6/12	16	Geforce 1050 Ti	4	Pascal / 6.1
ws	HP Z640 workstation	Xeon E5-1630	4/8	32	Tesla K40	12	Kepler / 3.5
HPC	Dell PowerEdge	Xeon Silver 4114	20/40	376	Tesla V100	16	Volta / 7.0
	C4140						

For “Speedup of ABEA algorithm”, time measurements were obtained by inserting *gettimeofday* timestamp function invocations directly into the C source code. Total execution time and the peak RAM usage in “Comparative performance of *f5c* with *Nanopolish*” sections were measured by running the *GNU time* utility with the *verbose* option.

## Results

### Speedup of ABEA algorithm

We initially compared the optimised GPU version with the optimised CPU version of the ABEA algorithm (not the unoptimised CPU version in the original *Nanopolish*, see below) by executing them on publicly available raw nanopore genome sequencing data. The CPU version was run with maximum supported threads on the tested systems. The optimised CPU version will be henceforth referred to as *C-opti* and the optimised GPU version will be referred to as *G-opti*.

**Fig. 3:**
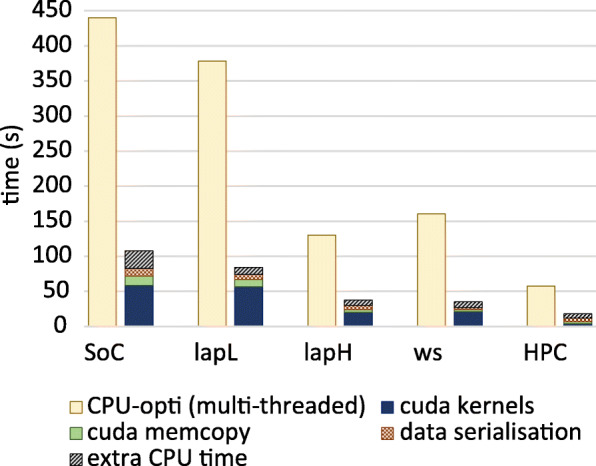
Performance comparison of ABEA on CPU vs GPU over a wide range of systems. Runtime for *C-opti* (left bars) and the *G-opti* (right bars). Runtime for the GPU has been broken down into: compute kernel time; different overheads (memory copying to/from the GPU, data serialisation time), and the extra CPU time due to CPU processing of the reads. The compute kernel time includes the sum of time for all GPU kernels. The extra CPU time is the additional time spent by the CPU to process *very long reads* and *ultra long reads* (see Supplementary materials) assigned to the CPU (excluding the processing time that overlaps with the GPU execution, i.e. only the extra time which the GPU has to wait after the execution is included)

First we benchmarked on five different systems (Table 2) over *D*_*small*_ dataset. Speedups (including all the overheads) observed for *G-opti* compared to *C-opti* are: ∼4.5× on the low-end-laptop and the workstation; ∼4× on Jetson TX2 SoC; and ∼3× on high-end-laptop and HPC (Fig. 3). Note that only a ∼3× speedup was observed on high-end-laptop and HPC (versus >=4 × on other systems) due to the CPU on those particular systems having a comparatively higher amount of CPU cores (12 and 40 respectively).

**Fig. 4:**
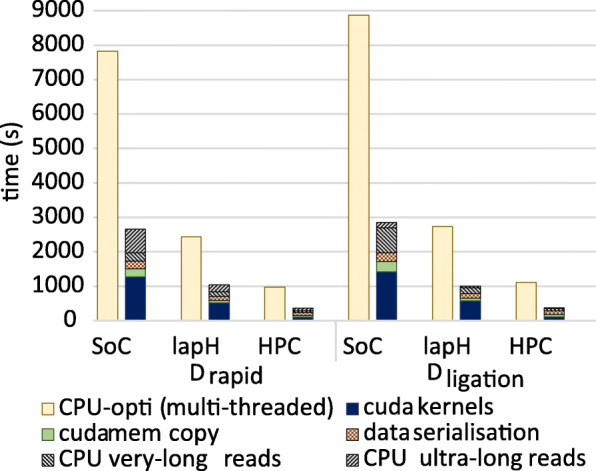
Performance comparison of ABEA on CPU vs GPU across over large datasets Runtime for *C-opti* (left bars) and the *G-opti* (right bars). Runtime for the GPU has been broken down into: compute kernel time; different overheads (memory copying to/from the GPU, data serialisation time), extra CPU time due to *very long reads* and *ultra long reads* (see Supplementary materials)

We next benchmarked on two larger datasets (*D*_*rapid*_ and *D*_*ligation*_). A speedup up of ∼3× was observed for all three systems for the two big datasets— *D*_*ligation*_ and *D*_*rapid*_ (Fig. 4). Due to more ultra long reads (>100kb) in *D*_*ligation*_ and *D*_*rapid*_ than in *D*_*small*_, the overall speedup for *SoC* is limited to around ∼3× compared to ∼4× for *D*_*small*_.

It is noteworthy to mention that comparing performance to the unoptimised CPU version in *Nanopolish* is not straightforward, as the time for individual components (e.g. ABEA) cannot be accurately measured because each read executes on its own code path (detailed in Supplementary Materials). We nonetheless estimated the runtime of unoptimised ABEA by injecting timestamp (*gettimeofday*) functions into the original *Nanopolish* code, directly before and after the ABEA component to measure runtimes for individual reads. *Nanopolish* was launched with multiple threads and the runtimes were averaged by the number of threads to get a reasonable estimate for ABEA. When evaluated using the *D*_*small*_ dataset, the optimised ABEA CPU version in *f5c* was ∼1.3-1.7 × times faster than the unoptimised ABEA in the original *Nanopolish* program (∼1.4× speedup on Jetson TX2, workstation and HPC, ∼1.7× on low-end-laptop and ∼1.3× on high-end-laptop).

### Comparative performance of *f5c* with *Nanopolish*

The overall performance of the GPU-accelerated ABEA algorithm was evaluated through a DNA methylation (5-methylcytosine) detection work-flow. We compared the total runtime for methylation calling using the original *Nanopolish* against *f5c* (both CPU-only and GPU-accelerated versions) by running on two publicly available nanopore datasets (see “Methods” section).

We refer to the original *Nanopolish* (version 0.9) as *nanopolish-unopti*, *f5c* run only on the CPU as *f5c-C-opti* and GPU accelerated *f5c* as *f5c-G-opti*. We executed *nanopolish-unopti*, *f5c-C-opti* and *f5c-G-opti* on the full datasets *D*_*rapid*_ and *D*_*ligation*_. Note that all execution instances were performed with the maximum number of CPU threads available on each system.

**Fig. 5:**
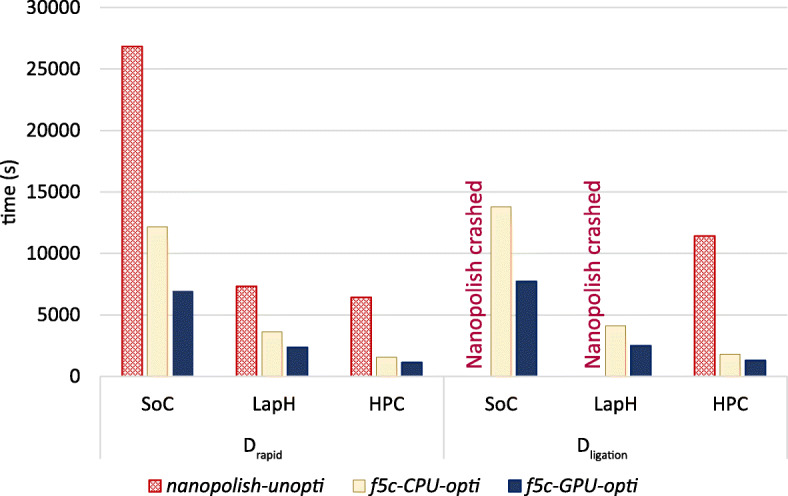
Comparison of *f5c* to *Nanopolish*. The reported run-times are for the complete methylation calling and also include disk I/O time

*f5c-C-opti* on the *D*_*rapid*_ dataset was: ∼2× faster than *nanopolish-unopti* on *SoC* and *lapH* and ∼4× faster on *HPC*. On *D*_*ligation*_, *nanopolish-unopti* crashed on *SoC* (limited by 8GB RAM) and *lapH* (16GB RAM) due to the Linux Out Of Memory (OOM) killer [12] (Fig. 5). On *D*_*ligation*_, *f5c-C-opti* on *HPC* was not only 6 × faster than *nanopolish-unopti*, but also consumed only ∼15 GB RAM, as opposed to >100 GB used by *nanopolish-unopti* (both with 40 compute threads). Hence, it is evident that CPU optimisations alone can do significant improvements.

When comparing the total execution time (including disk I/O) of the entire methylation calling process with different hardware acceleration options in *f5c*, *f5c-G-opti* was 1.7 × faster than *f5c-C-opti* on *SoC*, 1.5-1.6 × on *lapH* and <1.4× on *HPC* (Fig. 5). On *HPC*, the speedup was limited to <1.4× due to file I/O being the bottleneck. N.B. only the ABEA algorithm step utilises the GPU acceleration.

For the *D*_*rapid*_ dataset, the execution time of *f5c-G-opti* versus *nanopolish-unopti* was ∼4×, ∼3× and ∼6× faster on *SoC*, *lapH* and *HPC*, respectively (Fig. 5). On the *D*_*ligation*_ dataset on *HPC*, *f5c-G-opti* was a remarkable ∼9× faster.

Although parameters that may affect biological accuracy were untouched, we did observe subtle variations in the output as a consequence of hardware-based fluctuations in the treatment of floating point units. We assessed the impact of these subtle variations on the measurement of relative methylation frequencies by comparing results for *Nanopolish*, *f5c-C-opti* and *f5c-G-opti* on the *D*_*small*_ dataset, which encompasses 5M bases of human chromosome 20 with an average read coverage of 30 ×. Of the ∼32,000 surveyed CpG sites, *f5c-C-opti* and *f5c-G-opti* produced different methylation frequencies for only 6 (∼0.02%) and 65 (∼0.2%) positions, with an average position-specific difference in methylation frequency values of ∼1.5% and ∼0.4%, respectively. Both variants of *f5c* yielded overall Pearson correlation values of 0.99999 with *Nanopolish*. Moreover, the overall correlation between *Nanopolish* and bisulfite sequencing data from NA12878 is 0.88723, while the correlation for *f5c-C-opti* and *f5c-G-opti* is 0.88723 and 0.88724, respectively. The impact of hardware-based differences in the calculation of methylation frequencies is therefore negligible.

## Discussion and future work

High-throughput nanopore data analysis is a relatively new field that emerged with the release of the first ONT sequencing device (MinION) in 2014. Numerous nanopore data analysis algorithms have since been developed by biologists and bioinformaticians. However, work that explores computational bottlenecks, acceleration and parallisation techniques for such algorithms are limited, especially for those that exploit raw nanopore signal data, such as the ABEA algorithm.

There are a handful of methods that have been developed to accelerate the analysis of nanopore data. The proprietary base-calling software *Guppy* developed by ONT exploits NVIDIA GPUs for fast and accurate processing of raw nanopore data via deep neural networks [3]. Although the design details of *Guppy* are not publicly disclosed, they are likely to have benefited by a plethora of work focusing on GPU optimisations for neural networks. Another example of highly optimised software for third generation sequencing data is *minimap2*, a popular open source sequence aligner for long reads (including nanopore reads) that has recently been accelerated with the simultaneous use of GPUs and Intel Xeon Phi co-processors [13]. However, alignment in base-space is considerably different from signal-space, which is explored in this work. Recently, the NVIDIA corporation has shown an interest in developing open source libraries such as *Clara Genomics*[14] for accelerating long read data analysis on their GPUs. The *Clara Genomics* library contributes to nanopore data analysis domain through the acceleration of core algorithmic components such as all-vs-all read mapping and partial order alignments for genome assembly. Nonetheless, none of these algorithms focus on accelerating signal-space alignment.

A number of GPU accelerated versions of SW alignment have previously been reported [15–17]. However, differences between SW and ABEA significantly affect the efficient mapping of the algorithm and data structures to GPU architectures. For instance, band movement in ABEA during execution and random memory accesses to the pore-model in ABEA affect data dependencies (thus, the parallelism) and the memory layout (thus, the memory access patterns). Therefore, the GPU acceleration solutions proposed in these reports are ill-suited for ABEA. In addition, the above-mentioned works were developed for short, static read-lengths. Third generation sequencers produce variable long read lengths that vary significantly over a given dataset. Consequently, the strategies we disclose herein for efficient GPU memory allocation and load balancing are novel and significant improvements for ABEA.

**Fig. 6:**
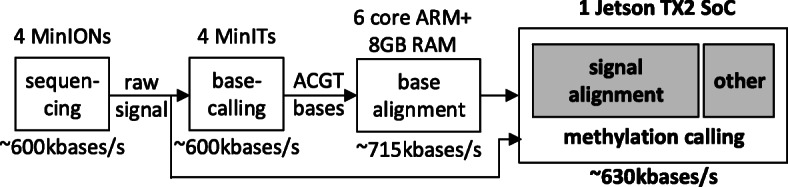
Human genome processing on-the-fly

Moreover, we demonstrate that a complete DNA methylation analysis of a human genome using raw Oxford *Nanopolish* sequencing data can be executed on an embedded system (e.g., a SoC equipped with ARM processor and an NVIDIA GPU) as shown in Fig. 6. The data processing speed is sufficient to keep up with data generated in real-time by four Oxford Nanopore MinION devices in parallel, or a GridION sequencer. GPU-enabled *f5c* can process such data using a single NVIDIA TX2 SoC, at a speed of >600 Kbases per second to keep up with the sequencing output (∼600 Kbases per second [18]), as shown in Fig. 6. Conversely, if the original *Nanopolish* was executed on the NVIDIA TX2 SoC, the processing speed is limited to ∼256 Kbases per second. The base-space alignment speed of ∼715 Kbases/s in Fig. 6 was obtained by running Minimap2 [19] on the Jetson TX2 with only 8GB using the partitioned-index approach we previously presented in [20].

It is also noteworthy to mention that we used *Nanopolish* v0.9 for the performance comparisons as subsequent releases of the software incorporated some of our optimisations, excluding those that did not require major code refactoring, such as GPU implementation, threading models and I/O processing interleaving. Furthermore, the optimisations we performed were focused on restructuring and fine-tuning the implementation of the algorithm to conform with computer hardware. Parameters that might affect the biological accuracy were untouched. Still, we extensively compared results of *f5c* with that of *Nanopolish* and verified that they are almost identical, greatly surpassing variation observed using alternative experimental approaches (i.e, bisulfite sequencing for 5mC detection).

Our work can not only reduce the hardware and bandwidth requirements for analysing raw Nanopore data, but can also improve the turnaround time for performing reference-guided raw nanopore signal processing, an analytic process that is used for base-calling and detecting non-standard nucleotides. In addition to embedded systems, our work benefits all computational systems, with or without GPU. For instance, our work enables methylation calling on laptops with <16GB of RAM. Furthermore, we have demonstrated that *a posteriori* methylation calling execution with *f5c* on high performance computers also benefits from a significant speedup.

## Conclusions

ABEA is a prominent bioinformatics algorithm for raw nanopore signal analysis. Although this algorithm is not massively parallel, we present a highly efficient implementation of ABEA that includes the (optional) use of GPUs. Through a number of memory optimisations and a heterogeneous processing strategy that uses both CPU and GPU, we were able to overcome several inherent challenges, such as prominent variations in sequencing read lengths. Our optimisations yield around 3-5 × performance improvement on a CPU-GPU system when compared to CPU only. We demonstrate that these optimisations are sufficient for the execution and completion of a DNA methylation detection workflow on an embedded SoC equipped with a hexa-core ARM processor and NVIDIA GPU (256 cores) in real-time. This work not only benefits embedded SoCs, but also a wide range of systems equipped with GPUs, from laptops to servers, as highlighted by a 9 × speedup and 6-fold memory reduction when performing methylation detection on a high-performance computing server. The source code of *f5c* is made available at https://github.com/hasindu2008/f5c.

## Supplementary information

**Additional file 1** Supplementary materials. PDF file that details all the supplementary materials.

## Data Availability

All data generated or analysed during this study are included in this published article and its supplementary information files. Source code of *f5c* is available in the GitHub repository, https://github.com/hasindu2008/f5c. A reproducible Code Ocean compute capsule is available at 10.24433/CO.3078978.v1

## Abbreviations

ABEA: Adaptive banded event alignment
CPU: Central processing unit
GPU: Graphics processing unit
SoC: System on chip
HPC: High-performance computer/computing
ONT: Oxford nanopore technologies
SW: Smith-waterman
SK: Suzuki-kasahara
POSIX: Portable operating system interface
CUDA: Compute unified device architecture
DRAM: Dynamic random access memory
RAM: Random access memory
GNU: GNU’s not unix
OOM: Out Of memory
