# Supplementary material for: GPU accelerated adaptive banded event alignment for rapid comparative nanopore signal analysis
Source: BMC Bioinformatics. 2020 Aug 5;21:343. doi: 10.1186/s12859-020-03697-x (PMC7430849; doi:10.1186/s12859-020-03697-x)
Supplement: Supplementary file 1 — Additional file 1 Supplementary materials. PDF file that details all the supplementary materials. [file 12859_2020_3697_MOESM1_ESM.pdf]

RESEARCH: SUPPLEMENTARY MATERIALS

Supplementary Materials: GPU Accelerated Adaptive Banded Event Alignment for Rapid Comparative Nanopore Signal Analysis

Hasindu Gamaarachchi[1,2*], Chun Wai Lam[1], Gihan Jayatilaka[3], Hiruna Samarakoon[3], Jared T Simpson[5,6], Martin A Smith[2,4] and Sri Parameswaran[1]

List of Tables

S1 Data arrays associated with ABEA and their sizes . . . 18
S2 GPU data arrays, pointer computation and heuristically determined sizes . . . 18
S3 measured quantities . . . 21
S4 adjustable user parameters . . . 21
S5 Information of the datasets . . . 26
S6 Different systems used for experiments . . . 26

List of Figures

S1 Illustration of a nanopore raw signal, events and *pore-model* . . . 5
S2 example nanopore read length distributions . . . 5
S3 Evolution of dynamic programming based sequence alignment . . . 7
S4 Adaptive Banded Event Alignment . . . 9
S5 Thread configuration of *pre-kernel* . . . 12
S6 Thread assignment of *pre-kernel*. The assignment for the first two reads are shown. Each thread block has a read assigned to it ($block_0$ refers to threads $t_{X=0,y=0}$ to $t_{x=WX-1,y=0}$, and $read_0$ is processed by all threads in $block_0$; similarly, $block_1$ refers to $t_{X=0,y=1}$ to $t_{x=WX-1,y=1}$ and $read_1$ is processed by threads in $block_1$). . . . 13
S7 Utility of *kcache* in the *core-kernel* to improve memory coalescing . . . 16
S8 Decision trees for resource optimisation 22
S9 Effect of individual optimisations . . . 27

List of Algorithms

S1 Adaptive Banded Event Alignment . . . 10
S2 Adaptive Banded Event Alignment - cell score computation . . . 10
S3 Adaptive Banded Event Alignment - log probability computation . . . 10
S4 Outline of execution flow . . . 12
S5 Adaptive Banded Event Alignment - *pre-kernel* . . . 14
S6 Adaptive Banded Event Alignment - *core-kernel* . . . 23
S7 Adaptive Banded Event Alignment - *core-kernel* - cell score computation . . . 23
S8 Adaptive Banded Event Alignment - *core-kernel* - log probability computation. . . . 23
S9 Memory allocation—data structure serialisation . . . 23
S10 heuristic memory allocation scheme . . . 24

Contents

S1 Detailed Background to ABEA 3
S1.1 Nanopore sequencing and analysis . . . 3
S1.1.1 Whole genome sequencing . . . 3
S1.1.2 DNA methylation . . . 3
S1.1.3 Nanopore sequencing and the raw signal . . . 3
S1.1.4 Nanopore read length distribution 4
S1.1.5 Sequence alignment/mapping in the base-space . . . 4
S1.1.6 Polishing/Downstream processing using raw signal . . . 4
S1.2 Methylation calling . . . 4
S1.3 Adaptive Banded Event Alignment (ABEA) 6
S1.4 GPU architecture and programming . . 11

S2 Extended Methodology 12
S2.1 Parallelisation and compute optimisations 12
S2.1.1 pre-kernel . . . 12
S2.1.2 core-kernel . . . 14
S2.1.3 post-kernel . . . 15
S2.2 Memory optimisation . . . 15

*Correspondence: hasindu@unsw.edu.au
[1]School of Computer Science and Engineering, University of New South Wales, Sydney, Australia
[2]Kinghorn Centre for Clinical Genomics, Garvan Institute of Medical Research, Sydney, Australia
Full list of author information is available at the end of the article

S2.2.1 Data array serialisation 15
S2.2.2 Heuristic based memory pre-allocation 17
S2.3 Heterogeneous processing 19
S2.3.1 Very long reads and ultra long reads 19
S2.3.2 Over segmented reads 20
S2.3.3 Batch size 20
S2.3.4 Detection of performance anomalies 20

S3 Extended Results **25**
S3.1 Experimental setup 25
S3.2 Effect of individual optimisations 25
S3.2.1 Compute optimisations 25
S3.2.2 Memory optimisations 25
S3.2.3 Heterogeneous processing 26

S4 Miscellaneous **26**
S4.1 Why Nanopolish had to be re-engineered? 26
S4.2 Additional advantages of *f5c* over *Nanopolish* 27

S1 Detailed Background to ABEA

Basic terms and concepts of DNA sequencing and data analysis are given in Section S1.1. Section S1.2 briefly explains methylation calling, an example nanopore data analysis workflow. Section S1.3 explains the Adaptive Banded Event Alignment (ABEA) algorithm, the algorithm which is optimised in this paper for execution on a CPU-GPU heterogeneous architecture. In Section S1.4, a brief account of GPU architectures and the programming methods for GPUs.

S1.1 Nanopore sequencing and analysis

S1.1.1 Whole genome sequencing

The *genome* is a long sequence composed of four types of nucleotide bases: adenine (A), cytosine (C), guanine (G) and thymine (T). Nucleotide bases will be simply referred to as *bases* hereafter. The human genome is around 3.2 gigabases (Gbases) long and is composed of 23 pairs of chromosomes (46 chromosomes in total), where each chromosome is a single molecule of continuous deoxyribonucleic acid (DNA) polymer. The process of reading strings of contiguous bases is called *sequencing*, and the resulting strings of bases are called *reads*. In order to be sequenced, DNA molecules must be extracted and purified from cells before being biochemically prepared for sequencing. This *library preparation* process can fragment chromosomes (especially large ones) into smaller segments–either intentionally or incidentally–which are 'read' by the sequencer. Given that samples contain multiple cells, and thus several distinct DNA molecules, and that sequencing may introduce errors, it is desirable to generate enough reads to cover a particular position several times. The average number of reads at a given position is termed sequencing *coverage*. High coverage facilitates the characterisation of genetic variation and correct for errors. A human genome sequenced at around 20× average coverage corresponds to around 64 Gbases of sequencing reads.

S1.1.2 DNA methylation

DNA undergoes naturally regulated biochemical modification through the addition of a methyl group to certain bases. Methylation is reversible and can control the activity of a DNA segment, such as turning the expression of genes on or off, without modifying the genetic code itself—a process called *epigenetic* regulation. DNA methylation is dynamically regulated during normal biological development and in function of environmental factors; it plays an important role in disease aetiology and clinical diagnostics [1, 2, 3]. Methylation of cytosine ('C') bases is of particular interest in human biology, where CpG dinucleotides ('C' base followed by a 'G' base) are dynamically methylated in normal development and disease [4, 5, 6].

S1.1.3 Nanopore sequencing and the raw signal

Nanopore sequencing is a third generation sequencing technology that involves physical observation of atomic properties of DNA fragments using a nanometer scale biological pore coupled to an ammeter. The pore acts as a bottleneck to generate characteristic disruptions in ionic current (in the range of pico-amperes) that are indicative of the molecules passing through the pore. The size and nature of the pore influence the measured instantaneous current and how it is subsequently analysed. Oxford Nanopore Technologies (ONT) sequencing devices measure DNA strand passing through biological nanopores composed of recombinant (or 'designer') proteins at an average speed of ~450 bases/s while the current is sampled and digitised at ~4000 Hz[1]. The instantaneous current measured in ONT nanopore depends on 5-6 contiguous bases [7]. The measured signal also presents stochastic noise due to several factors, such as homopolymers (same base repeating multiple times) which produce constant current levels, contaminants in the sample, entanglement of long DNA strands, depletion of ions, etc [8]. Additionally, the movement speed of the DNA strand through the pore can vary, causing the signal to warp in the time domain [8]. The raw signal is converted into character representations of DNA bases (e.g. A,C,G,T) using artificial neural networks, generating a typical accuracy >90% for single reads [9]. This conversion process is referred to as *base-calling* and the software tools that perform this conversion are referred to as *base-callers*. Please refer to [7] for a detailed discussion of ONT sequencing.

An example of a raw nanopore signal is shown in Fig. S1a using the blue coloured line. Assume that the signal is generated from the DNA sequence *GAATACGAAAATCATTA* which passed through the nanopore. In this example, the instantaneous current of the signal is affected by a string of 6 contiguous bases, known as a *6-mer* (or a *k-mer* in general). Let us assume that the annotation of the signal to the corresponding *k-mers* is known (the process of getting this annotation is detailed in Section S1.2). The *6-mers* in the sequence and the corresponding segments in the raw signal are marked using vertical grey lines in Fig. S1a. When the DNA sequence *GAATACGAAAATCATTA* moves through the pore, the first *6-mer* is *GAATAC*. Similarly, the subsequent *6-mers* are *AATACG*, *ATACGA*, *TACGAA*, ..., *TCATTA*. *True annotation* (depicted by dotted green coloured step function in Fig. S1a) corresponds to the *ideal* average level of current for each *k-mer*. These ideal average values are obtained

[1]these are typical values at present which may vary in the future

using the *pore-model* provided by ONT, which is elaborated in Section S1.2. The red coloured step function corresponds to an *event*—detailed in Section S1.2.

To deduce the sequence from the *k-mers*, the base at the centre (3rd base) of each k-mer is taken, as shown on the bottom of Fig. S1a. For instance, we take A from $GAATAC$, T from $AATACG$, C from $TACGAA$, etc. Hence, we obtain a sequence $ATACGAAAATCA$ which is a part of the original sequence $GAATACGAAAATCATTA$. Note that the beginning and the end of the sequence (GA at the beginning and TTA at the end) are clipped.

S1.1.4 Nanopore read length distribution

The length of the reads generated from nanopore sequencers can vary from several hundred bases to even more than 2 million bases. A typical sequencing run of a particular sample (which completes after 48-64 hours) generates millions of such reads. The distribution of the read lengths varies in function of DNA integrity, extraction protocols, and sample preparation methods. Example distributions for three different samples are shown in Fig. S2, where both x and y axes are in logarithmic scale. The average read length of a sample typically falls between 8-20 Kilobases.

S1.1.5 Sequence alignment/mapping in the base-space

Once a nanopore read is base-called, the sequence is aligned to a reference sequence. A reference sequence consists of a previously generated consensus sequence (such as the human genome reference). Sequence alignment involves global optimisation algorithms to identify the most similar target and to compare any differences between sequences. Compared to biologically occurring variation in individual genomes (<1% difference to the reference), the error-rate of nanopore sequencing is relatively high (5-10%). Thus, sequence alignments derived from nanopore reads are distinct in nature from previous sequencing technologies (such as highly accurate short reads). Consequently, unique analytic tools must be considered when aligning such reads. Alignment tools such as Minimap2 [10] that employ a hash table based genome index followed by a base-level dynamic programming alignment step can successfully align long and noisy reads.

S1.1.6 Polishing/Downstream processing using raw signal

The base-space alignment discussed previously in Section S1.1.5 is followed by 'polishing', a downstream processing step that utilises both the base-space alignment results and the raw signal. The polishing step reuses the raw signal to recover the lost biological information during base-calling. This polishing step can be to correct errors during base-calling or to detect modified nucleotide bases (eg: DNA methylation).

Previous research has shown that identification of genetic variants can be improved up to an accuracy of more than 99% by using raw signal data from multiple overlapping reads [11, 12]. Thus, the downstream analysis that reuses raw signal data could correct for base-calling errors. It has also been shown that methylated C bases can be differentiated from non-methylated C bases by the use of signal data, using algorithms such as the one implemented in the software package *Nanopolish* [13]. Thus, the downstream analysis that reuses raw signal data could detect modified nucleotide bases.

Signal-space alignment is one of the crucial steps performed in these downstream analyses such as error correction and modified base detection. This signal alignment step is described in the context of modified base detection in the following sections.

S1.2 Methylation calling

As discussed above, important biological information is lost during base-calling. Some base-calling models may not accommodate methylated data, either because they are trained on unmethylated sequences, or because they abstract away non-canonical bases. Therefore, these molecules may be erroneously classified as unmethylated bases. The process of identifying methylation is known as *methylation calling.*

As implemented in *Nanopolish*, methylation calling requires: 1, raw signals; 2, base-called reads; and 3, base-space alignment to a reference genome (output of the sequence alignment step described above). For a given read, the main steps for methylation calling are: 1, event detection; 2, signal-space alignment; and 3, Hidden Markov Model (HMM) profiling. These steps are performed for each individual read in the data set.

Event detection is the time series segmentation of the raw signal based on sudden signal level changes. Each segment is called an *event* and is typically denoted using the mean ($\mu_{\bar{x}}$), standard deviation ($\sigma_{\bar{x}}$) and the duration of the raw signal samples ($n_{\bar{x}}$) pertaining to the particular segment. The red step function in Fig. S1a denotes such detected events by plotting the mean value of the samples ($\mu_{\bar{x}}$) corresponding to the segment. Note that in Fig. S1a, events (red line) roughly match to the true annotation (dotted green line), nevertheless, are not exactly the same. Mostly, the signal has been over-segmented (eg: portion corresponding to k-mer $CGAAAA$ has been segmented into 3 events) and seldom under-segmented (eg: k-mer $AAATCA$).

To obtain the true annotation in Fig. S1a, the events detected in the event detection step are aligned to a generic k-mer model signal. This generic k-mer model

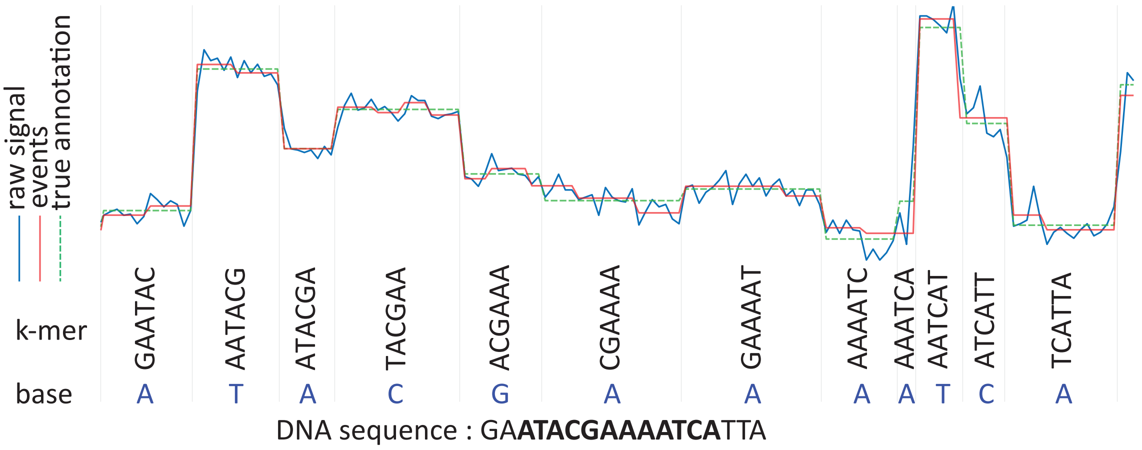

(a) An example nanopore raw signal and events

**k-mer**	**mean**	**sd**
AAAAAA	μ_0	σ_0
AAAAAC	μ_1	σ_1
AAAAAG	μ_2	σ_2
AAAAAT	μ_3	σ_3
AAAACA	μ_4	σ_4
.	.	.
.	.	.
.	.	.
.	.	.
TTTTTT	μ_{4095}	σ_{4095}

(b) an example *pore-model*

Figure S1: Illustration of a nanopore raw signal, events and *pore-model*

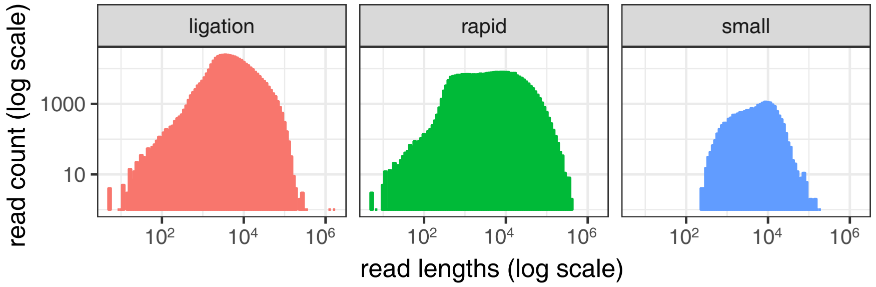

Figure S2: example nanopore read length distributions

signal is derived from the base-called sequence and a *pore-model* provided by ONT. The *pore-model* corresponds to a table of all possible k-mers matched to their mean signal value and standard deviation (4^6 k-mers if k is 6, as shown in Fig. S1b)[2]. For each 6-mer

[2]there can be other values in addition to mean and standard deviation, which are not required for our methylation calling

in the base-called read, the corresponding entry in the *pore model* (*mean,sd*) is obtained and these *mean,sd* pairs form the generic k-mer model signal. *Nanopolish* aligns the events from the event detection step to this generic k-mer model signal by using the algorithm named *Adaptive Banded Event Alignment (ABEA)* explained in Section S1.3.

ABEA above produces the alignment between the events and the k-mers in the base-called read. The base-space sequence alignment then is used to deduce which event corresponds to a given k-mer in the reference genome. Finally, this alignment between the events and the k-mers in the reference genome are subjected to Hidden Markov Model (HMM) profiling to identify if a given base is methylated or not.

S1.3 Adaptive Banded Event Alignment (ABEA)

Algorithms to determine the optimal alignment between two biological sequences typically utilise dynamic programming (DP). Very first of such algorithms, Needleman–Wunsch (NW) algorithm dates back to the 1970s. NW and its variant, Smith–Waterman (SW) algorithm are of quadratic time and space complexity. Both NW and SW were used extensively to perform fine alignment of DNA sequences with high quality. However, due to its extended time consumption, several heuristic improvements were made to improve the speed of alignment without losing quality.

Fig. S3a exemplifies an original SW based alignment (no heuristic) between two sequences, *target sequence* $t_0t_1t_2t_3t_4t_5$ *(6 bases long), and query sequence* $q_0q_1q_2q_3q_4q_5q_6q_7$ (8 bases long). The DP table (scoring matrix) contains 6x8 cells as shown. First, the initial values are set (shown as 0 in the figure); second, the score for each cell ($s_{x,y}$) is computed based on a scoring scheme; and third, the trace-back (backtracking denoted by red arrows on the figure) starting from the highest scoring cell and ending at a cell with 0 score, outputs the optimal alignment that yields the highest score (please refer [14] for a detailed explanation of SW).

In the case of short read alignment, the sequences to be aligned are small (typically 100-500 bases). Two sequences (each sequence ~100 bases long) can be aligned by filling ~10^4 cells. While a single such alignment can be quickly handled by a modern computer, it is very computationally demanding when the number of alignments to be performed scales up to hundreds of millions (or billions), which is the case for short reads. To reduce the number of computations, banded alignment approaches were introduced, where only the cells in the DP table along the left diagonal band are computed as shown in Fig. S3b. The underlying assumption is that, the sequences that are aligned to each other are essentially similar, thus the alignment (the trace-back arrows) should lie close to the left diagonal. Note that in the figure, only the cells in a band of width (W) four have been computed, yet has been sufficient to contain the alignment inside the band.

In contrast to short reads, long reads which emanate from Nanopore, PacBio, etc. have lengths which are 100 to 1000 orders of magnitude bigger than short reads, are noisier (with a greater number of errors) and are typically not suitable for such small static bands. The 10% base-calling error rate would result in the alignment significantly deviating from the diagonal. A major advantage of long reads is the detection of long indels (insertions and deletions occasionally spanning lengths longer than short reads themselves). When aligning such reads, the alignment path deviates significantly from the diagonal. The high errors and the large indels require the bands to be of large width if they are to be static.

High band-width requirement causes processing times to be extremely high when aligning millions of reads. To improve the speed of this processing, Suzuki-Kasahara (SK) heuristic algorithm [15] was introduced in 2017. SK utilises an adaptive band scheme, letting a smaller band to contain such an alignment within the band, which is exemplified below.

Consider the same example in Fig. S3b (performed previously with a static band of size 4) is now performed only with a band-width of size 3, as shown the Fig. S3c. Observe that the band is no longer sufficient to contain the whole alignment, i.e. the cell $s_{4,7}$ which previously contained the maximum score is no longer computed, thus the trace-back would begin from the maximum value within the band, which leads to a non-optimal alignment. This is remedied using an *adaptive band* in Fig. S3d. The band moves either down or to the right (the band dynamically adapts) as determined by the Suzuki-Kasahara heuristic, which is illustrated by blue arrows. Observe how the alignment is possible to be contained inside a band of width 3 which was previously infeasible using a static band.

Modified versions of the SK algorithm are used for event-space alignment as exemplified in *Nanopolish* and is referred to as *Adaptive Banded Event Alignment (ABEA)*. In ABEA, the events are aligned to the k-mers of the base-called read (as stated in Section S1.2). As typically there are many more events than k-mers (usually by a factor 1.5-2) due to the frequent over-segmentation of events (discussed in Section S1.2), event alignment is even more difficult than base-space long read alignment if performed with static banding around the diagonal. Thus, an adaptive band is essential for event alignment.

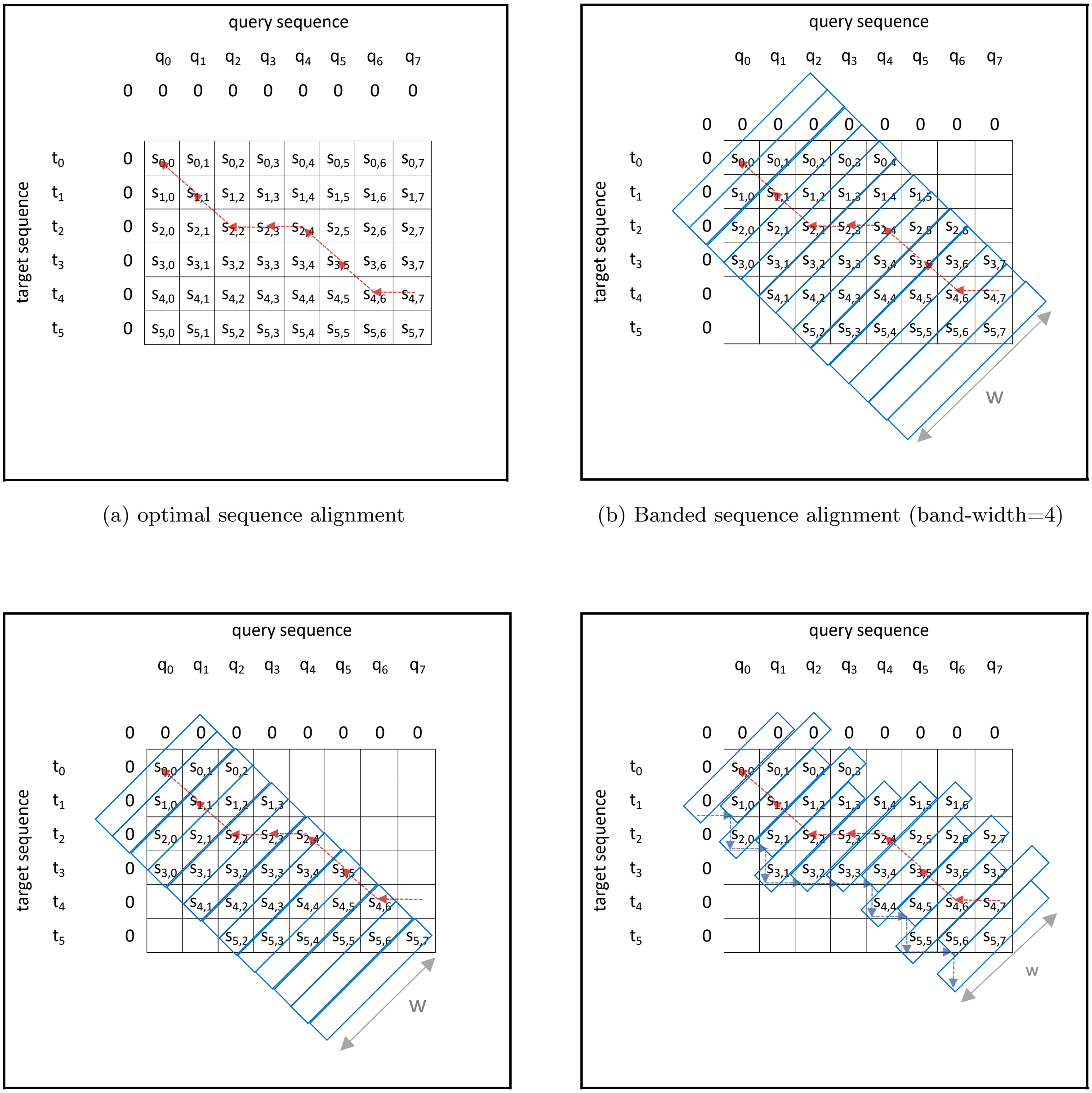

(a) optimal sequence alignment

(b) Banded sequence alignment (band-width=4)

(c) Banded sequence alignment (band-width=3)

(d) Adaptive banded sequence alignment

Figure S3: Evolution of dynamic programming based sequence alignment

The scoring function for signal alignment uses a 32 bit floating point data type, as opposed to 8-bit integer data type in sequence alignment. Furthermore, the signal alignment scoring function that computes the log-likelihood (which we elaborate shortly) is computationally expensive.

A simplified example of ABEA is shown in Fig. S3e. In Fig. S3e the horizontal axis represents the events (results of the event detection step) and the vertical axis represents the *ref* k-mers (k-mers of the basecalled read). The dynamic programming table (DP table) in Fig. S3e is for 13 events, indexed from e_0-e_{12} vertically, and the *ref* k-mers, indexed from k_0-k_5 horizontally. As mentioned previously for computational and memory efficiency, only the diagonal bands (marked using blue rectangles) with a band-width of

W (typically W=100 for nanopore signals) are computed. The bands are computed along the diagonal from top-left (*b0*) to bottom-right (*b17*). Each cell score is computed in function of five factors: scores from the three neighbouring cells (up, left and diagonal); the corresponding *ref* k-mer; and, the event (shown for the cell e_6, k_3 via red arrows in Fig. S3f, details of the computation is explained later). Observe that all the cells in the nth band can be computed in parallel as long as the $n-1^{th}$ and $n-2^{th}$ bands are computed beforehand. To contain the optimal alignment, the band adapts by moving down or to the right as shown using blue arrows in Fig. S3e. The adaptive band movement is determined by the Suzuki-Kasahara heuristic rule [15].

Algorithm S1 summarises the ABEA algorithm used in *Nanopolish* [13] and is explained with the aid of the example in Fig. S3e.

The input to the Algorithm S1 are: 1, *ref* (the sequenced read in base-space—eg: *GAATACG...*); 2, *events* (the output of the event detection step mentioned in Section S1.2); and 3, *model* (*pore-model*—Fig. S1b). As mentioned in Section S1.2, the ABEA algorithm (Algorithm S1) attempts to align the events to the generic signal model (produced with the use of *ref* and the *model*) and outputs the alignment as *event-ref* pairs. The algorithm requires three intermediate arrays, namely *score* (2D floating point array), *trace* (2D byte array) and *ll* (1D pointer array) to formulate the intermediate state during alignment computation, which is the DP table shown in Fig. S3e). Note that, *ll* stands for lower-left, which holds the coordinate of the start point of the band.

The initialisation of the first two bands (*b0* and *b1*) in Fig. S3e is performed by line 20 of Algorithm S1. Then, the outer loop (starting from line 3) iterates through rest of the bands from top-left to bottom-right of the DP table. The inner loop (lines 11-15) iterates through each cell in the current band *bi*. To ensure that only cells within the DP table are computed, the loop counter j iterates from min_j to max_j, instead of 0 to $W-1$. Lines 4-9 of Algorithm S1 correspond to the movement of the band (corresponds to the blue arrows in Fig. S3e). Band movement is actuated by proper placement of the band in the static 2D arrays, *score* and *trace* via the array *ll* using the functions *move_ band_ right* and *move_ band_ down*.

Line 12 of the algorithm performs the cell score computation (explained in detail later) and generates a score and a direction flag for subsequent backtracking, which are henceforth stored in the arrays *score* and *trace*. When all the cells in the DP table are computed, the final operation is to find the actual alignment (*event-ref* pairs) through the backtracking operation (line 17 of Algorithm S1 and red trace-back arrows in Fig. S3g), which uses both the cell scores and the direction flags stored in *trace*.

The *compute* function (called at line 12 of Algorithm S3e) is elaborated in Algorithm S2. A number of heuristically determined constants suitable for Nanopore data, which are used during subsequent calculations are listed at the beginning of this algorithm. The first step of this algorithm is the computation of *lp_ emission*, a log probability value (likelihood of the particular signal event being the particular *ref* k-mer), performed using the function elaborated in Algorithm S3. This computed *lp_ emission* is used in lines 4-5 of Algorithm S2 along with the heuristically determined constants (*lp_ skip,lp_ stay,lp_ step*) to compute three scores from the diagonal, left and up (*score_ d, score_ u, score_ l*). The maximum of the three scores and direction from which the max score came (flags pertaining to diagonal, up or left) are returned as outputs from this function. Line 3 of Algorithm S2 refers to accessing the scores of the upward, left and diagonal cells which were previously mentioned with respect to cell e_6,k_3 and the red arrows in Fig. S3f.

The log probability computation in Algorithm S3 involves floating point log probability computations. For the k-mer at the specific *ref* position, the *pore-model* table (Fig. S1b) is accessed to obtain the corresponding model values. This *model_ kmer* (mean and the standard deviation of the particular model k-mer) and the mean value of the event is used for the log probability computation as shown in Algorithm S3. Note that for event alignment neither the standard deviation or the duration of the event are used.

The above elaboration covers the ABEA algorithm to a sufficient level to explain our GPU implementation and optimisations. Therefore, implementation details of checking out-of-bounds array accesses and the backtracking process were not discussed. Furthermore, the concepts of 'trim state' and 'event scaling' were not discussed as the control flow of the algorithm is not affected by them. Thus, those details are not vital for the elaboration GPU implementation. However, for the sake of completeness, a brief account of 'trim state' and 'read-model scaling' are given below.

The raw signal may contain samples at the beginning or the end that may be ignored by the base-caller and hence does not contribute to the base-called sequence. These samples may be open-pore signals immediately before or after the DNA molecule is detected (i.e. the electric current when nothing is in the nanopore), or perhaps part of the adaptor (molecules bounds to the ends of the DNA molecules to enable sequencing). The 'trim states' allow the alignment to ignore these samples, since such samples should not be considered to be part of the base-called read.

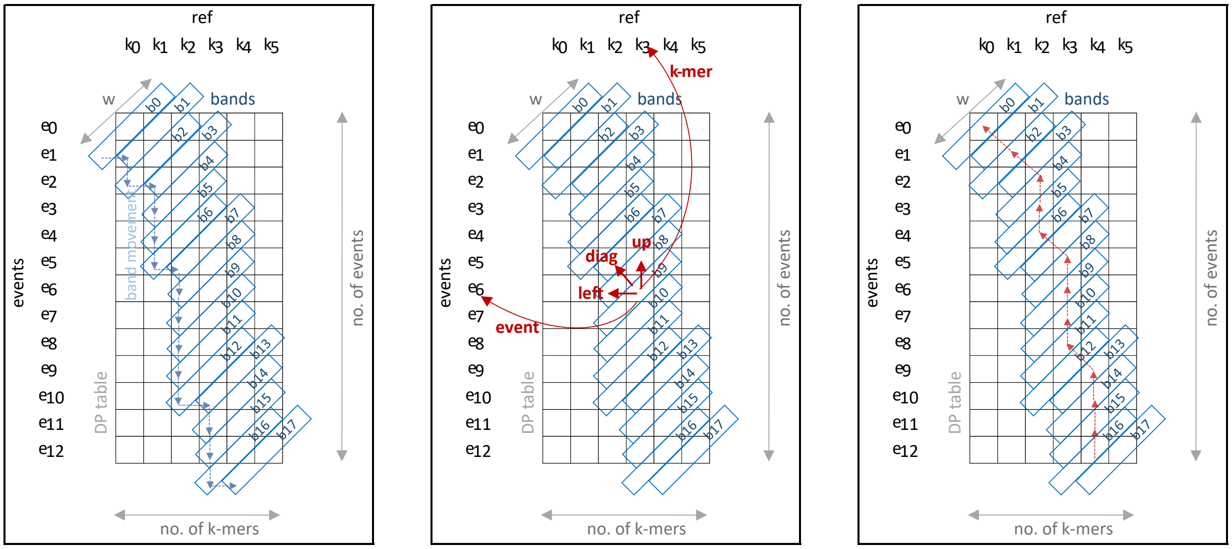

(e) band movement (f) computing a single cell score (g) trace-back

Figure S4: Adaptive Banded Event Alignment

Due to slight variations between different nanopores and characteristic changes of the same nanopore with time, an event will not directly match the *pore-model* in Fig. S1b [16]. Therefore, to account for these variations either the events or the *pore-model* should be scaled on a per-read basis. In *Nanopolish*, two scaling parameters namely *shift* and *scale* are estimated on a per-read basis, prior to ABEA algorithm, using a 'Method of Moments' approach [16]. Then, during ABEA, the *pore-model* mean values are scaled using these two parameters. The scaling should be performed at line 5 of Algorithm S3 as $\mu \leftarrow model\_kmer.mean \times scale + shift$ instead of directly assigning $model\_kmer.mean$ to μ.

Algorithm S1 Adaptive Banded Event Alignment

Input:
ref[] : the base-called read (1D char array)
model : *pore-model* (Fig. S1b)
events[] : event table containing $\{\mu_{\bar{x}},\sigma_{\bar{x}},n_{\bar{x}}\}$ of each event—1D {*float,float,float*} array

Output:
alignment[] : alignment denoted by a list of {*event index,k-mer index*}—1D {*int,int*} array

Intermediate:
score[][] : scores of the cells in banded area—2D float array
trace[][] : back-track flags of the cells in banded area—2D char array
ll_idx[] : {event index,k-mer index} for each band's lower left cell—1D {*int,int*} array

1: **function** *align*(*ref,model,events*)
2: *initialise_first_two_bands(score,trace,ll_idx)* ▷ band b0 and b1 in Fig. S3e, see line 20
3: **for** *i* ← *2 to n_bands* **do** ▷ Iterate from b2 to b17 in Fig. S3e
4: *dir* ← *suzuki_kasahara_rule(score[i-1])* ▷ *score[i-1]* is of the previous band
5: **if** $dir == right$ **then**
6: *ll_idx[i]* ← *move_band_to_right(ll_idx[i - 1])* ▷ see line 28
7: **else**
8: *ll_idx[i]* ← *move_band_down(ll_idx[i - 1])* ▷ see line 33
9: **end if**
10: *min_j,max_j* ← *get_limits_in_band(ll_idx[i])* ▷ get index bounds in current band*
11: **for** *j* ← *min_j* to *max_j* **do** ▷ Iterates through each cell in band i
12: *s,d* ← *compute(score[i-1],score[i-2],ref,events,model)* ▷ see Algorithm S2
13: *score[i,j]* ← *s*
14: *trace[i,j]* ← *d*
15: **end for**
16: **end for**
17: *alignment* ← backtrack(*score, trace. ll*) ▷ the trace-back red arrows in Fig. S3g.
18: **end function**
19:
20: **function** *initialise_first_two_bands*(*score,trace,ll_idx*)
21: *score[0,*], trace[0,*]* ← $-\infty, 0$ ▷ Initialise first band b0
22: *score[1,*], trace[1,*]* ← $-\infty, 0$ ▷ Initialise second band b1
23: *ll_idx[0]* ← $\{ei_0,ki_0\}$ ▷ $ei_0 = 1$ and $ki_0 = -1$ in Fig. S3e**
24: *ll_idx[1]* ← $\{ei_1,ki_1\}$ ▷ $ei_1 = 1$ and $ki_1 = 0$ in Fig. S3e**
25: *score[0,*si_0] ← 0 ▷ si_0 is 0 is Fig. S3e***
26: **end function**
27:
28: **function** *move_band_to_right*(*ll_previous*)
29: *ll_current.event_idx* ← *ll_previous.event_idx + 1*
30: *ll_current.kmer_idx* ← *ll_previous.kmer_idx*
31: **end function**
32:
33: **function** *move_band_down*(*ll_previous*)
34: *ll_current.event_idx* ← *ll_previous.event_idx*
35: *ll_current.kmer_idx* ← *ll_previous.kmer_idx+1*
36: **end function**

*For instance, in Fig. S3e *min_j=1,max_j=1* for b0 and b17; *min_j=0,max_j=1* for b1; *min_j=1,max_j=2* for b16; and, *min_j=0,max_j=2* for the rest

**these initial event and k-mer indices corresponding to the lower left of the band are computed with respect to band-width W

***the score of cell that corresponds to k-mer index -1 in band b0 is initiliased to 0

Algorithm S2 Adaptive Banded Event Alignment - cell score computation

Constants:
$events\_per\_kmer = \frac{n\_events}{n\_kmers}$
$\epsilon = 1^{-10}$
lp_skip $= \ln(\epsilon)$
lp_stay $= \ln(1 - \frac{1}{events\_per\_kmer+1})$
lp_step $= \ln(1.0 - e^{lp\_skip} - e^{lp\_stay})$

1: **function** COMPUTATION(*score_prev,score_2ndprev,ref,events,model*)
2: *lp_emission* ← *log_probability_match(ref,events,model)* ▷ see Algorithm S3
3: *up,diag,left* ← *get_scores(score_prev,score_2ndprev)* ▷ see red arrows in Fig. S3f
4: *score_d* ← *diag* + *lp_step* + *lp_emission*
5: *score_u* ← *up* + *lp_stay* + *lp_emission*
6: *score_l* ← *left* + *lp_skip*
7: *s* ← *max(score_d,score_u,score_l)*
8: *d* ← *direction from which the max score came*
9: **end function**

Algorithm S3 Adaptive Banded Event Alignment - log probability computation

1: **function** LOG_PROBABILITY_MATCH(*ref,events,model*)
2: *event,kmer* ← *get_event_and_kmer(ref,events)* ▷ see red arrows in Fig. S3f
3: x ← *event.mean*
4: *model_kmer* ← *get_entry_from_poremodel(kmer,model)*
5: $\mu \leftarrow model\_kmer.mean$
6: $\sigma \leftarrow model\_kmer.stdv$
7: $z \leftarrow \frac{x-\mu}{\sigma}$
8: *lp_emission* ← $\ln(\frac{1}{\sqrt{2\pi}}) - \ln(\sigma) - 0.5z^2$
9: **end function**

S1.4 GPU architecture and programming

Graphics Processing Units (GPUs) were originally designed as co-processors for graphics processing and rendering. Graphics processing and rendering algorithms involve pixel-wise operations that expose fine-grained parallelism, thus GPUs consist of hundreds of compute cores to perform parallel processing. Eventually, the concept of general purpose graphics processing units (GPGPU) emerged where the GPUs were exploited to accelerate compute intensive, yet highly parallelism portions of general purpose algorithms. GPUs are quite popular in scientific computations due to the significant speedup when used for common matrix manipulation which contains fine-grained parallelism. From around a decade ago, GPUs which are explicitly designed for high performance computers are available (e.g., Tesla GPUs from NVIDIA).

GPUs are of Single Instruction Multiple Data (SIMD) architecture (or more accurately Single Instruction Multiple thread, as stated by NVIDIA), where multiple threads run the same stream of instructions in parallel yet on different data. Conversely, CPUs are of Multiple Instruction Multiple Data (MIMD) architecture, where each thread runs its own instruction sequence and own data stream, independent of the others. GPUs have hundreds or even thousands of processing cores while a CPU would maximally have a few dozen cores. However, the GPU cores are relatively less complex (fewer instructions, smaller caches, no sophisticated branch prediction units, etc.) and run at a lower clock speed when compared to a CPU. Due to these significant differences between CPU and GPU architectures, serial algorithms designed and developed for the CPUs are not suitable for execution on GPUs. Such algorithms have to be adapted and parallelised in a way that the GPU architectural features are efficiently used.

NVIDIA provides a programming model/framework for programming their GPUs for general purpose computations, called Compute Unified Device Architecture (CUDA). CUDA includes CUDA C/C++ (extended C/C++ syntax) and an Application Programming Interface (API) to provide a platform to write programs for the NVIDIA GPU. We used this CUDA C/C++ for our GPU implementation of the Adaptive Banded Event Alignment algorithm.

We will now briefly give GPU/CUDA related terms. Readers are advised to refer to [17] and [18] for further information.

A GPU *kernel* is a function that is executed on a GPU. A GPU kernel is written from the execution perspective of a single GPU thread. These GPU kernels will run in parallel, based on the parameters specified with the function call, known as the *thread configuration*. This thread configuration in CUDA is an abstraction which employs a hierarchy of threads. In the thread hierarchy, a group of threads is known as a *block*. A group of blocks forms a *grid*. Instances of a single kernel are executed in a single grid. Blocks and grids can be 1 dimensional, 2 dimensional or 3 dimensional. The presence of this thread hierarchy lets the programmer organise and map the threads conveniently to a grid. These logical threads would be mapped to the hardware cores automatically by the underlying driver software and hardware.

A thread block consists of one or more *thread warps*. A warp is a group of threads sharing the same program counter. A data dependent conditional branch inside a warp causes the threads to execute each code path while disabling threads that are not in the path, known as *warp divergence*. The warp divergence affects the performance and should be minimised.

The *occupancy* is the percentage of the number of active warps to the maximally supported warps on the GPU. A lesser occupancy leads to underutilisation of GPU resources. Thus, a higher occupancy is preferable for better utilisation of GPU resources.

GPUs also employ a memory hierarchy. Relatively larger but slow Dynamic Random Access Memory (DRAM) that forms the lowest level in the memory hierarchy is known as *global memory*. Global memory is typically allocated using *cudaMalloc()* API function. Memory allocated in this global memory can be exclusively accessed by all the threads in the grid. The next level in the memory hierarchy which is made of relatively fast, yet smaller SRAM is called *shared memory*. Shared memory is allocated on a per-thread-block basis and is shared by all the threads in the block. Shared memory can be called user managed cache (more accurately a programmer managed cache) as the programmer is expected to identify and load frequently accessed data to the shared memory. In addition, there are one or more levels of SRAM caches managed by the hardware. The registers are the fastest and highest in the hierarchy and are allocated by the compiler on a per-thread basis.

The global memory can be easily saturated when hundreds of threads compete to access the memory at the same time. Thus, memory accesses should be batched such that contiguous threads access contiguous memory locations. This process is referred to as *memory coalescing* and reduces global memory requests thus reducing the impact on performance compared to scattered memory accesses. Additionally, the programmer could utilise the shared memory to load and store frequently accessed data, which also reduces global memory traffic.

Algorithm S4 Outline of execution flow

```
for batch of n reads do
    ...                       ▷ CPU processing steps before the Adaptive Banded Event Alignment eg: event detection
    memcpy_ram_to_gpu(...)    ▷ copy inputs of the Adaptive Banded Event Alignment to the GPU memory
    gpu_alignment(...)        ▷ Perform the event alignment on the GPU
    memcpy_gpu_to_ram(...)    ▷ copy results back to the RAM
    ...                       ▷ CPU processing steps after the alignment eg: HMM
end for
```

S2 Extended Methodology

To optimise the performance on GPUs, we process a batch of reads (original source code processes a read at a time) at a time. Such batch processing minimises data transfer initialisation overhead (between RAM and GPU memory); reduces the GPU kernel invocation overhead; and, allows parallelism which sufficiently occupies all available GPU cores. The execution flow follows the typical GPU programming paradigm, which is elaborated in Algorithm S4. In Algorithm S4, *gpu_alignment(...)* refers to the GPU implementation of the Adaptive Banded Event Alignment (CPU algorithm is elaborated in Algorithm S1). We present our methodology in three steps: parallelisation and compute optimisations in Section S2.1; memory optimisation in Section S2.2; and, the resource optimisation through heterogeneous processing in Section S2.3.

S2.1 Parallelisation and compute optimisations

The GPU implementation of the Adaptive Banded Event Alignment (ABEA) algorithm is broken into three GPU kernels. Breaking down into the three GPU kernels allows for efficient thread assignment based on the workload type, synchronisation of all GPU threads (a GPU kernel execution is inherently a synchronisation barrier [17]) and minimising warp divergence compared to a big all-in-one GPU kernel.

The three GPU kernels are:

- *pre-kernel* - Initialising the first two bands of the dynamic programming table (corresponds to line 2 of algorithm S1) and pre-computing frequently accessed values by the next GPU kernel;
- *core-kernel* - The filling of dynamic programming table which is the compute intensive portion of the ABEA algorithm (corresponds to line 3-16 of Algorithm S1 composed of the nested loop); and,
- *post-kernel* - Performs backtracking (corresponds to line 17 of algorithm S1)

S2.1.1 pre-kernel

The *pre-kernel* initialises the first two bands of the dynamic programming table (initialisation performed at line 2 of Algorithm S1 on CPU). The *pre-kernel* also pre-computes the values in a data structure called *kcache*, a newly introduced data structure in the GPU implementation that improves cache hits during the subsequent execution of the *core-kernel*.

A simplified version of the *pre-kernel* is in Algorithm S5 and thread configuration for the invocation of the *pre-kernel* is in Fig. S5. Note that the GPU kernel is presented (as is always the case) from the perspective of a single GPU thread in Fig. S5.

Each cell in Fig. S5 represents a GPU thread denoted as t, where the subscripts x and y denote the thread index along the x-axis and the y-axis respectively. The thread grid in Fig. S5 is composed of n thread blocks, where n is the number of reads in the batch. Each thread block contains WX threads where WX is the nearest upper ceiling multiple of 32 to the band-width W (band-width of the ABEA algorithm); i.e. $WX = (int)\frac{W+31}{32} \times 32$ For instance, if W=*100*, WX is 128. The reason for taking a multiple of 32 is due to performance attributed by a thread block size that a multiple of the warp size (warp size is 32 currently) [18]. As shown in Fig. S5, a single thread block composed of WX threads is assigned to a single read.

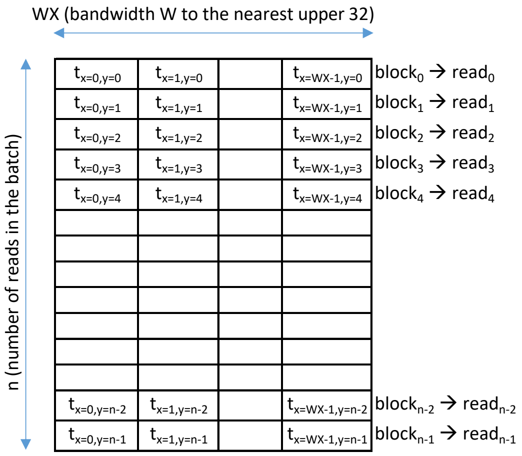

Figure S5: Thread configuration of *pre-kernel*

In the Algorithm S5, lines 2-3 get the thread index of the thread being executed, i.e. the thread indices denoted as x and y in Fig. S5. Line 4 obtains the memory pointers of the input array *ref*; intermediate arrays *score* and *trace*; and the *kcache*, the use in which is explained in the memory optimisation Section (Section S2.2).

Lines 5-8 of Algorithm S5 initialises the first two bands of the dynamic programming table (which was performed at line 2 of original CPU Algorithm S1). The kernel is in from the perspective of a single thread

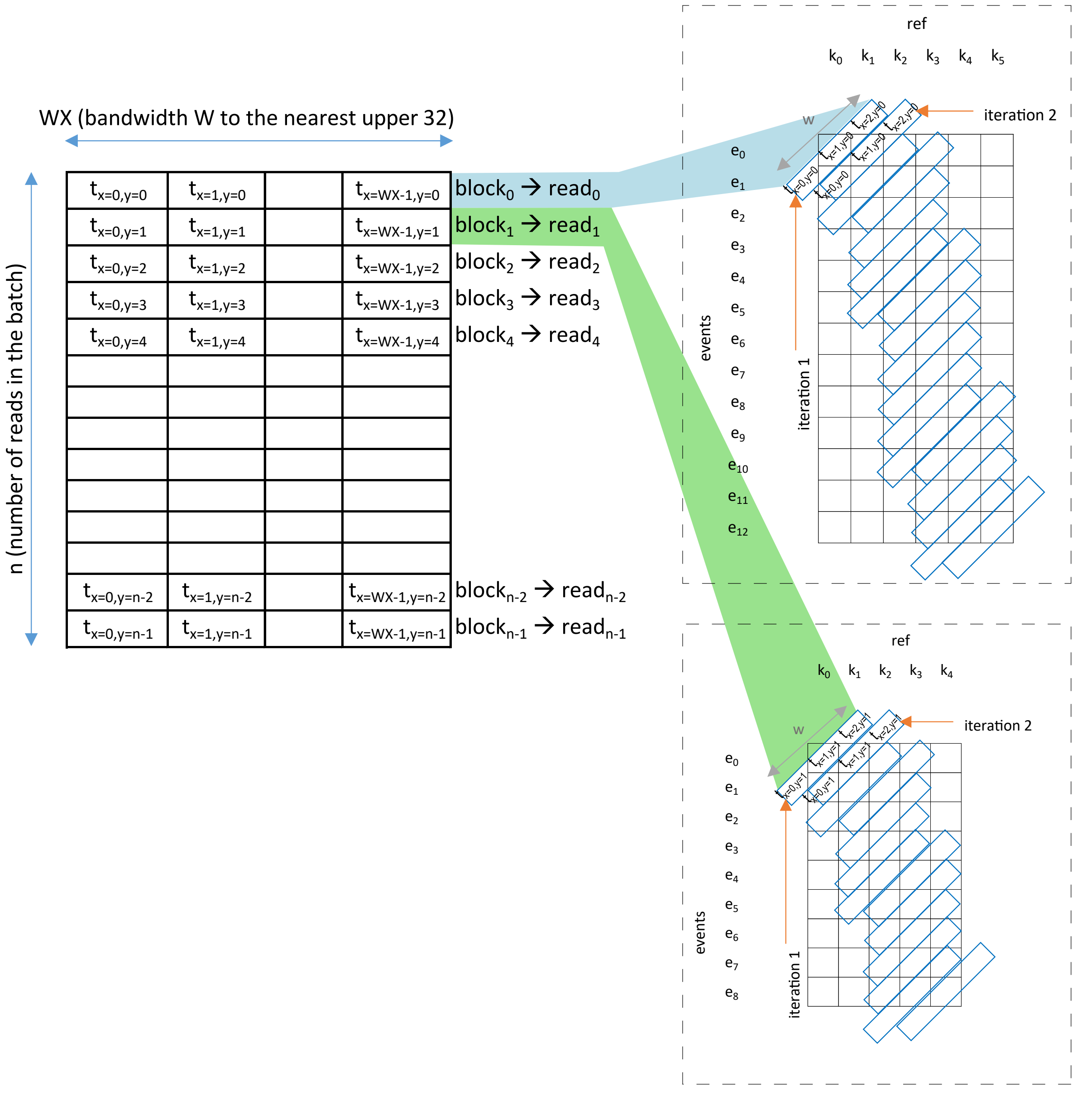

Figure S6: Thread assignment of *pre-kernel*. The assignment for the first two reads are shown. Each thread block has a read assigned to it (block$_0$ refers to threads $t_{X=0,y=0}$ to $t_{x=WX-1,y=0}$, and read$_0$ is processed by all threads in block$_0$; similarly, block$_1$ refers to $t_{X=0,y=1}$ to $t_{x=WX-1,y=1}$ and read$_1$ is processed by threads in block$_1$).

and thus a single cell is initialised by a single thread. The collective execution of all the threads in Fig. S5, effectively sets a band for all the reads in the batch in parallel, which is illustrated in Fig. S6. Note that, only the first two reads are elaborated in Fig. S6, and in reality each thread block has a read assigned to it. In Fig. S6, each cell in band$_0$ (marked as iteration 1) contains the index of the thread which performs the initialisation at line 6 of Algorithm S5. Similarly, iteration 2 corresponds to line 7 of Algorithm S5.

Algorithm S5 Adaptive Banded Event Alignment - *pre-kernel*

1: **function** *align_pre(...,model)* ▷ ... refers to other arguments which are later explained Section S2.2
2: *j* ← *thread index along x* ▷ the x subscript of a thread Fig. S5
3: *i* ← *thread index along y* ▷ the y subscript of a thread Fig. S5
4: *(ref,score,trace,ll_idx,kcache)* ← *get_cuda_pointers(i,...)* ▷ get memory pointers of the arrays corresponding to read i (explained in Section S2.2)
5: **if** $j < W$ **then** ▷ Though a block is WX wide (Fig. S5) only W threads should execute
6: *score[0,j], trace[0,j]* ← $-\infty, 0$ ▷ corresponds to line 21 of Algorithm S1
7: *score[1,j], trace[1,j]* ← $-\infty, 0$ ▷ corresponds to line 22 of Algorithm S1
8: **end if**
9: **if** *j==0* **then** ▷ only thread 0 process this Section
10: *ll_idx[0]* ← $\{ei_0, ki_0\}$ ▷ corresponds to line 23 of Algorithm S1
11: *ll_idx[1]* ← $\{ei_1, ki_1\}$ ▷ corresponds to line 24 of Algorithm S1
12: *score[0,*si_0*]* ← 0 ▷ corresponds to line 25 of Algorithm S1
13: **for** *k=0 to numkmers* **do** ▷ Iterate through each kmer in ref from left to right
14: *kmer* ← *get_kmer_at(ref,k)* ▷ k-mer at position k in *ref*
15: *kcache[k]* = *get_entry_from_poremodel(kmer,model)*
16: **end for**
17: **end if**
18: **end function**

The *if* condition on line 5 of Algorithm S5 is to limit the threads to the width of the band W, a consequence of selecting WX which is a multiple of 32 (as stated previously). Note that there is a 1024 thread limit for a block [17] in current NVIDIA CUDA/GPU architecture, thus our implementation will only work for a maximum band-width of 1024. This limit is more than sufficient for a typical W of 100 in ABEA.

Lines 10-11 of Algorithm S5 initialises the index of the lower left band which corresponds to lines 23-24 of Algorithm S1. Note that this initialisation is executed by one thread per read (thread id 0 along y-axis). Lines 13-16 in Algorithm S5 initialises *kcache*. As stated previously *kcache* is a newly introduced array for the GPU implementation to minimise random accesses to the GPU memory during the *core-kernel* and will be explained in Section S2.1.2. Note that, this *kcache* initialisation in lines 13-16 is also executed by one thread per read (thread id 0 along y-axis). The loop in 13-16 can be further parallelised; however, as the time spent on *pre-kernel* is comparatively negligible (see results), further parallelising this loop is superfluous.

S2.1.2 core-kernel

A simplified version of the *core-kernel* which fills the dynamic programming table in Fig. S3e (corresponds to lines 3-16 of the original Algorithm S1) is in Algorithm S6. This kernel is executed with the same kernel thread configuration as *pre-kernel* in Fig. S5. Thus, a batch of reads is processed in parallel with a block of threads assigned to a single read in a similar way to that in *pre-kernel* (Fig. S6). The only difference in Fig. S6 for the *core-kernel* is that the third band to the last band are processed instead of the first two bands.

All the W cells in a given band (Fig. S3e) are computed by W number of GPU threads in parallel (lines 26-30 of Algorithm S6), thus the inner loop of Algorithm S1 (lines 11 and 15) is now no longer present. However, the outer loop of Algorithm S1 cannot be parallelised due to band n depending on $n-1$ and $n-2$ bands as explained in the background. The movement/placement of the band (described in background) is performed by a single thread using the condition given on line 13 Algorithm S6 that limits the code segment to thread 0. In addition, synchronisation barriers per-thread-block basis (*__syncthreads*) in Algorithm S6 prevent any data hazards due to multiple threads assigned to a single read.

Another notable difference in the GPU implementation is the use of GPU shared memory [17] (user-managed cache or more accurately programmer-managed cache) for exploiting the temporal locality in the memory accesses to the dynamic programming table (n^{th} band in Fig. S3e is computed using bands *n-1* and *n-2*). Shared memory is allocated for three bands (current, previous band and second previous) by line 6-7 of Algorithm S6 which are then initialised at lines 9-10 of Algorithm S6. These initialised memory locations are used during band direction computation (lines 14-21 of Algorithm S6) and the cell score computation (lines 27-28 of Algorithm S6), eliminating any accesses to the slow GPU global memory (shared memory-SRAM vs global memory-DRAM). The cell score is written to the global memory at the end of the iteration (line 32 of Algorithm S6) as scores are later required for backtracking. Finally, current, previous and second previous bands are set for the next iteration (lines 33-36 of Algorithm S6).

As stated under Section S2.1.1, the data structure *kcache* introduced to the GPU implementation facilitates memory coalescing by minimising random memory accesses to the *model* array (*pore-model* array in Fig. S1b). If *kcache* did not exist, access pattern by contiguous threads in the *core-kernel* (shown for the iteration 5 of read 0) would look like in Fig. S6a where accesses to the *ref* are shown in green colour arrows and the subsequent accesses to the *pore-model* are in red colour arrows. The green arrows (relates to getting the k-mer at line 2 of Algorithm S3 in the CPU version) are spatially local and would facilitate memory

coalescing in the GPU. However, red arrows (relates to line 4 of Algorithm S3 in the CPU version) to the *model* array are random accesses. Note that such random accesses would occur during each iteration (iteration 3 to the last band iteration). Such multiple threads accessing random GPU memory locations degrade the performance due to smaller and less powerful GPU caches (compared to CPU), for instance, 32KB *pore model* array is larger than 8KB GPU constant cache [17].

These random accesses are eliminated by the *kcache* constructed in *pre-kernel* (stated under Section S2.1.1) which is then passed as an argument to the *compute* function at line 27 in Algorithm S6). This *kcache* is then passed on to the *log_probability_match* function (at line 2 of Algorithm S7) which is then used at line 4 of Algorithm S8. The construction of the caches in the *pre-kernel* requires random accesses to the model as shown in Fig. S6b, which happens only once. However, this *kcache* is utilised by the *core-kernel* in every iteration and facilitates memory coalescing (see green arrows in Fig. S6c which are spatially local accesses to the *kcache* by contiguous threads in iteration 5).

It is noteworthy to mention that allocating one thread block per read is critical (in the kernel configuration) to: use lightweight block synchronisation primitives *__syncthreads* (instead of expensive kernel invocations as synchronisation barriers [17]); minimise warp divergence (otherwise the longest read in the thread block would consume the longest time which corresponds to the band filling loop); and, use shared memory per read (shared memory is allocated per block).

S2.1.3 post-kernel
The backtracking operation performed by this *post-kernel* (one thread assigned to one read) does not expose fine grained parallelism as in previous kernels and thus not ideal for the GPU. However, performing this on GPU is still advantageous when compared to transferring huge intermediate arrays (*scores* and *trace*—size in the order of GB) from GPU to the RAM. In addition, no additional memory in the RAM is required, thus reducing peak RAM usage.

Allocating one thread block per read (as in *core-kernel* to reduce warp divergence) is not ideal for this *post-kernel* due to the lack of fine grained parallelism (i.e. 1 block having 1 thread), which results in reduced GPU occupancy (occupancy will be limited by the maximum thread blocks that can simultaneously reside in a GPU multi-processor). This is remedied without affecting the warp divergence by allocating a large number of threads per block (eg: 1024) and then limiting only the first thread in the warp (a warp is composed of 32 contiguous threads [17] and thus thread with indices 0, 32, 64, 96, ... , etc.) to perform the actual computation (backtracking for a read).

S2.2 Memory optimisation
CPU version of the Adaptive Banded Event Alignment (ABEA) algorithm performs dynamic memory allocations (*malloc*) on a per read basis. The number of reads in a dataset is in the order of millions and thus incur millions of *malloc* calls. However, dynamic memory allocations (*malloc* performed inside GPU kernels) are extraordinarily expensive in terms of execution time [17]. In-fact, our initial GPU kernel implementation which performed such memory allocations was more than 100× slower than the CPU implementation. An intuitive approach of statically allocating memory at the compile time is not practical as nanopore read lengths vary significantly (~100 bases to >1 Mbases as explained previously) and thus the associated data structures vary from ~200 KB to >1.5 GB. We present a methodology that significantly reduces the number of memory allocations by pre-allocating large chunks of contiguous memory at the beginning of the program to accommodate a batch of reads, which are then reused throughout the life-time of the program. The sizes of these large chunks are determined by the available GPU memory and the average number of events per base (i.e. average value of the number of events divided by the read length). For a given batch of reads, we assign reads to the GPU until the allocated GPU memory chunks saturate, and the rest of the reads are assigned to the CPU.

We describe the memory allocation technique in two steps: in Section S2.2.1 how the memory allocation for a batch of reads at a time is performed; and, in Section S2.2.2, how the method in Section S2.2.1 can be expanded to reuse large chunks of memory, allocated at the beginning of the program.

S2.2.1 Data array serialisation
In the three GPU kernels elaborated in Section S2.1, the associated data arrays per each read are *ref*, *kcache*, *events*, *score*, *trace*, *ll_idx* and *alignment* (final output from the *post-kernel*). If any of these arrays are allocated inside the GPU kernels on a per-read basis, for instance if *score* and *trace* arrays are allocated at line 4 of Algorithm S5 using *malloc*), the performance will be degraded.

We identified that the sizes of all the aforementioned data arrays are dependent only on the read length (known at run-time during file reading) and the number of events for the read (known after event detection described in Section S1). Thus, the sum of read lengths and the number of events for a batch of n reads (GPU

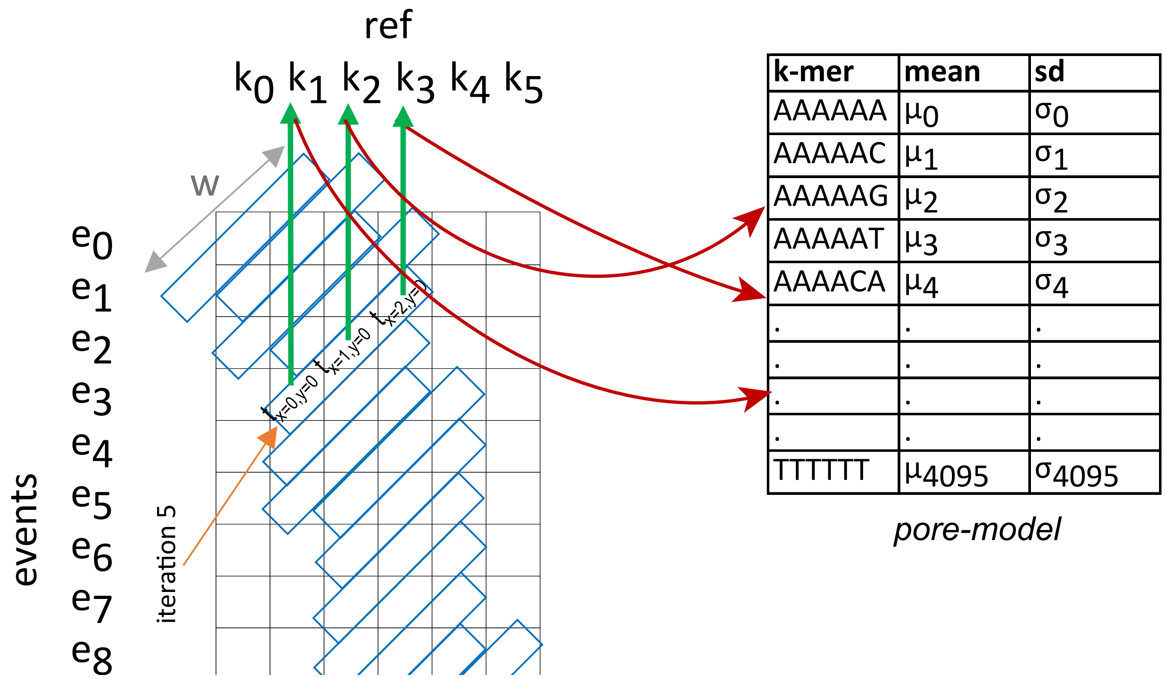

(a) Random accesses to the *model* array (red arrows) when *kcache* is not employed

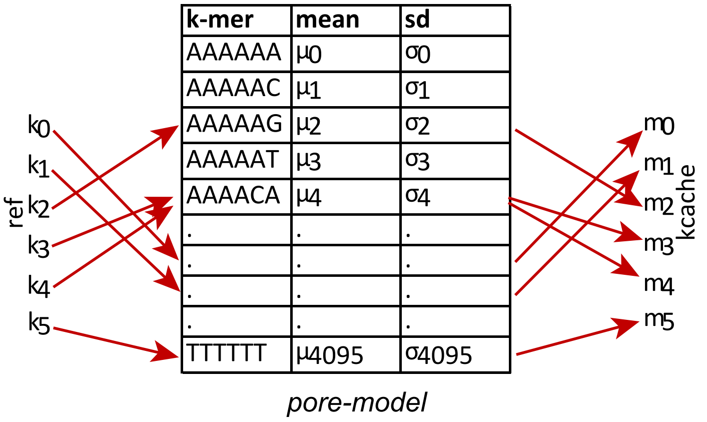

(b) Construction of the *kcache* in *pre-kernel*

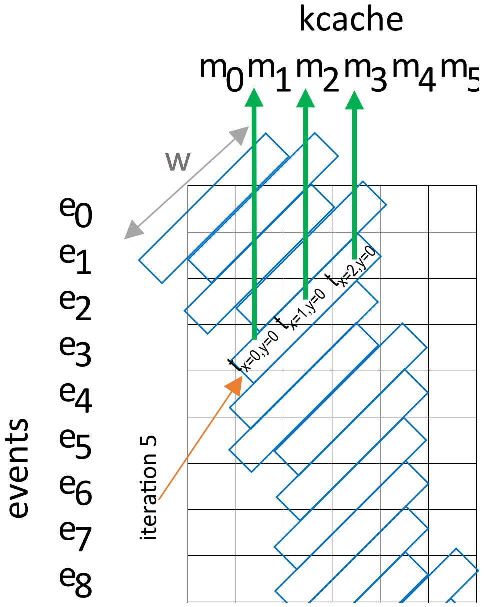

(c) Spatially local memory accesses (green arrows) when *kcache* is employed

Figure S7: Utility of *kcache* in the *core-kernel* to improve memory coalescing

processes a batch of n reads at a time) is used to calculate the sizes of memory allocations required for the particular batch according to the formulation below.

Let n be the number of reads loaded to the RAM (from the disk) at a time. Let $r[]$ be the read length and $e[]$ be the number of events for all the reads in batch

of n reads. Column 1 of Table S1, lists the data arrays. The size of arrays *ref* and *kcache* depends only on read lengths r; *events* and *alignment* depend on the number of events e; and, *score*, *trace* and *ll_idx* depend on both read length r and number of events e. Based on these dependencies, the arrays are categorised in Table S1 by horizontal separators. The second column of Table S1 states the data-type size of each array, denoted by constants of the form c_x. Typical values of these constants (in our implementation) are given inside the brackets. For instance, the data type for *ref* is *char* and thus C_r is 1 byte. The data type for events is a *struct* of size C_e that is 20 bytes. Note that, the exact values may depend on the implementation and the underlying processor architecture, nevertheless are constants known at compile time. The third column of Table S1 shows the size required for the particular array for a single read, i.e. the size for the ith read (assume 0 based index origin) in the batch of n reads. For instance, *ref* depends on the read length of the particular read and the datatype, thus the size is $C_r r[i]$. *Score* depends on read length, number of events, data type size and band-width (W), thus $WC_s(r[i]+e[i])$. The last column of Table S1 is the total size required for a batch of reads (based on the sum of r and e). For instance, the sum of all the *ref* arrays for the batch is the product of data type size C_r and sum of all read lengths in the batch $\sum_{i=0}^{n-1} r[i]$.

Based on the total array sizes in the last column Table S1, we can allocate seven big chunks of linear contiguous memory in the GPU. Let the base address of those chunks be represented by uppercase letters: REF; $KCACHE$; $EVENTS$; etc. These memory allocations are performed using *cudaMalloc()* API calls, just before the kernel invocations and are deallocated after the kernels. Note that for now, we do these allocations and deallocations for each batch of reads.

The GPU arrays *REF*, *KCACHE*, *EVENTS*, etc. allocated using *cudaMalloc* above are 1D arrays, thus multi-dimensional arrays in the RAM (eg: an array of pointers—each pointer pointing to a string/char array) must be serialised/flattened. One option is to save a series of pointers associated with each above array during the serialisation and then utilising those pointers for addressing a particular element later. However, this can be performed better by storing only two offset arrays of length n each: *read offset* array $p[]$, which is the cumulative sum of read lengths in the batch ($p[i] = \sum_{j=0}^{i-1} r[j]$); and, *event offset* array q, which is the cumulative sum of events in the batch ($q[i] = \sum_{j=0}^{i-1} e[j]$). Note that, r and e have the same definitions as before. These two offset arrays p and q can be used to deduce the associated pointer to a given element when required, by computing the array offset as shown in Table S2a. The first column of Table S2a is the base address of the large GPU arrays we allocated above. The offset of the element pertaining to the ith read (assume 0-indexing) in the particular array is given in the second column of Table S2a. The definition of constants C_x and W are the same as for the previous Table S1. These 1D array base addresses in the first column of Table S2a and the two associated offset arrays $p[]$ and $q[]$, are passed as arguments to the GPU kernels (Algorithm S5 and Algorithm S6). These arguments are used for the memory pointer computation inside the GPU kernels (line 4 of Algorithm S5 and line 4 of Algorithm S6) based on the second column of Table S2a.

Algorithm S9 elaborates how the above mentioned strategy is integrated into the previous execution flow depicted in Algorithm S4. Lines 3-7 of Algorithm S9 show how the offset arrays p and q are computed for each batch of reads. Line 8 of Algorithm S9 performs the serialisation of the multi-dimensional arrays with the use of offset arrays p and q. Line 9 of Algorithm S9 allocates GPU arrays based on sizes in the last column of Table S1. Then, the serialised arrays are copied to allocated GPU memory (line 10 of Algorithm S9), GPU kernels (the three kernels discussed in Section S2.1) are executed (line 11) and the alignment result is copied back from the GPU (line 12). At the end, the alignment result is converted back to multi-dimensional arrays (line 13) and then the GPU memory (allocated at line 9) is deallocated (line 14).

The offset arrays p and q (and also REF, KCACHE, EVENTS, etc.) are passed onto the GPU kernels and are utilised inside the GPU kernels to compute the memory pointers (line 4 of Algorithms S5 and S6) through the equations listed on the second column of Table S2a.

The limitation of this strategy is the GPU memory allocation and de-allocation (line 9 and 14 of Algorithm S9) performed for each batch of reads (which is expensive on certain GPUs, see Section S3.2.2). This limitation is remedied by the heuristic based pre-allocation strategy explained in the next subsection.

S2.2.2 Heuristic based memory pre-allocation

The GPU memory allocations in the previous section which were performed for each batch could be eliminated by pre-allocating all the available GPU memory at the startup of the program and then re-using for subsequent batches of reads). If the sizes of the arrays depended only on the read length, the total read length accommodable into the available GPU memory can be derived. Then, the available memory can be allocated among the seven large arrays (REF, $KCACHE$, $EVENTS$, etc.) in the correct proportion. However, these array sizes depend both on the

Table S1: Data arrays associated with ABEA and their sizes

**Array**	**Data type size (bytes)**	**Size for read i in batch**	**Size per batch**
ref[]	C_r (1)	$C_r r[i]$	$C_r \sum_{i=0}^{n-1} r[i]$
kcache[]	C_k (12)	$C_k r[i]$	$C_k \sum_{i=0}^{n-1} r[i]$
events[]	C_e (20)	$C_e e[i]$	$C_e \sum_{i=0}^{n-1} e[i]$
alignment[]	$C_a(8)$	$2C_a e[i]$	$2C_a \sum_{i=0}^{n-1} e[i]$
score[][]	C_s (4)	$WC_s(r[i] + e[i])$	$WC_s \sum_{i=0}^{n-1} (r[i] + e[i])$
trace[][]	C_t (1)	$WC_t(r[i] + e[i])$	$WC_t \sum_{i=0}^{n-1} (r[i] + e[i])$
ll_idx[]	$C_l(8)$	$C_l(r[i] + e[i])$	$C_l \sum_{i=0}^{n-1} (r[i] + e[i])$

Table S2: GPU data arrays, pointer computation and heuristically determined sizes

(a) Computation of pointer for the read i

**1D GPU array (base address)**	**Offset to element i in the batch**
REF	$C_r p[i]$
KCACHE	$C_k p[i]$
EVENTS	$C_e q[i]$
ALIGNMENT	$2C_a q[i]$
SCORE	$WC_s(p[i] + q[i])$
TRACE	$WC_t(p[i] + q[i])$
LL_IDX	$C_l(p[i] + q[i])$

(b) Heuristic allocation

**1D GPU array (base address)**	**Allocated size per batch**
REF	$C_r X$
KCACHE	$C_k X$
EVENTS	$C_e Y$
ALIGNMENT	$2C_a Y$
SCORE	$WC_s(X + Y)$
TRACE	$WC_t(X + Y)$
LL_IDX	$C_l(X + Y)$

read length and the number of events that are unknown at the beginning of the program; thus, memory cannot be partitioned among the data arrays. Therefore, We present a heuristic approach that exploits characteristics of nanopore data to estimate the proportion to maximally utilise the available GPU memory. In summary, we obtain the average number of events per base (average of the number of events divided by read length), use this average to determine the maximum read length that can be accommodated to the GPU, and proportionally allocate the GPU arrays. This approach is formulated as follows.

The sum of all the cells in column 4 of Table S1 is total memory required for a batch of n reads. This sum simplifies to equation 1 (due to the properties of constants) where $C_R = C_r + C_k + WC_s + WC_t + C_l$ and $C_E = C_e + 2C_a + WC_s + WC_t + C_l$. This sum represents the total size of all array (for adapted banded event alignment algorithm) for a batch of n reads.

$$S = C_R \sum_{i=0}^{n-1} r[i] + C_E \sum_{i=0}^{n-1} e[i] \tag{1}$$

If $\bar{\mu}$ is the average number of events per base (total number of events divided by the total read length for all reads in the batch), we can write as $\sum_{i=0}^{n-1} e[i] = \bar{\mu} \sum_{i=0}^{n-1} r[i]$. Now substituting this in equation 1 gives $S = (C_R + \bar{\mu} C_E) \sum_{i=0}^{n-1} r[i]$. We observed that for a sufficient batch size (>64), $\bar{\mu}$ is stable ~2.5 (on more than 10 datasets we tested). Let this estimated value for $\bar{\mu}$ be represented by the constant μ. Thus, the total memory required for a batch of reads can be estimated using equation 2.

$$M = (C_R + \mu C_E) \sum_{i=0}^{n-1} r[i] \tag{2}$$

Equation 2 can be used to estimate the maximum number of bases (sum of read lengths) that a given amount of GPU memory can accommodate. Let M in equation 2 be the available GPU memory. Then, the approximate maximum number of bases X that fits available GPU memory M can be computed via equation 3. Then, the associated total number of total events Y that the GPU memory can accommodate, is found by equation 4.

$$X = floor\left(\frac{M}{C_R + \mu C_E}\right) \tag{3}$$

$$Y = floor(\mu X) \tag{4}$$

These X and Y allow the available GPU memory to be allocated among the seven large arrays (REF, $KCACHE$, $EVENTS$, etc.) with approximately correct proportions, as shown in the second column of Table S2b. The values in the second column of Table S2b are obtained by substituting $\sum_{i=0}^{n-1} r[i]$ with X and $\sum_{i=0}^{n-1} e[i]$ with Y in the last column of Table S1.

By incorporating the above heuristic based memory allocation strategy to Algorithm S9, we get the execution flow in Algorithm S10. The major changes to the previous Algorithm S9 are highlighted in blue text. Now the GPU memory is allocated at the beginning of the program based on the estimated X and Y on line 1 of Algorithm S10. As X and Y are approximations, the GPU arrays may saturate for certain batches of reads. Line 6 of Algorithm S10 checks if GPU arrays are saturated and assigns the read to either GPU (line 9) or CPU (line 11), accordingly. Only a few reads are assigned to the CPU and these few reads are processed on the CPU in parallel to the GPU kernel execution, and thus no additional execution time is incurred.

With the heuristic based memory pre-allocation strategy described in this section, *cudaMalloc* operations are invoked only at the beginning of the program and thus no additional memory allocation overhead during the processing. Note that, our implementation is future proof; i.e. μ is a user specified parameter (that is initialised to 2.5 by default) in case nanopore data characteristics change in the future.

S2.3 Heterogeneous processing

If all the reads were of similar length, GPU threads that process the reads would complete approximately at the same time, and thus GPU cores will be busy throughout the execution. However, as stated in Section S1, there can be a few reads which are significantly longer than the other reads (we will refer to them as *very long reads*). When the GPU threads process reads in parallel, the presence of such *very long reads* will cause all other GPU threads to wait until the GPU threads processing the longest read complete. This thread waiting leads to the underutilisation of GPU cores. Thus, we process these *very long reads* on the CPU while the GPU is processing the rest in parallel. However, there can be exceptionally long reads (we will refer to them as *ultra long reads*) which the CPU would take longer time than what the GPU took to process the whole batch. Such reads would lead the GPU to idle until the CPU completes. Thus, *ultra long reads* will be skipped and will be processed separately at the end by the CPU. Similarly, there can be a few *over segmented reads* (i.e. reads with a significantly higher events per base ratio than the others) which cause GPU underutilisation. These over-segmented reads will also be processed on the CPU.

We discuss these problems of *very long reads* and *ultra long reads* in detail with examples in Section S2.3.1, along with the solutions. Then, in this Section S2.3.2, we discuss the problem of over segmented reads and the respective solution. Then, in Section S2.3.3, we discuss another factor that affects performance, the batch size (number of reads loaded to the RAM at a time). Finally, in Section S2.3.4, we describe a method to detect and prompt the user of any drastic impacts on performance along with suggestions to tune parameters to minimise the impact.

S2.3.1 Very long reads and ultra long reads

Consider a batch of reads where ~90% of the reads are less than 30 Kbases in length. Assume the longest read in the batch is 90 Kbases. Assume that the GPU is processing all the reads (in the batch) in parallel. Suppose that GPU threads processing reads of length <30 Kbases (90% of the threads) would complete in <300ms while GPU threads processing the longest 90 Kbases read would take 900ms. As a result, the completed GPU threads will have to wait for additional 600ms. Similarly, the few *very long reads* consume a significant time to process on the GPU in comparison to other reads in the batch. The majority of the GPU threads will have to wait and this causes underutilisation of GPU compute-cores. Furthermore, *very long reads* negatively affects the GPU occupancy by occupying a significant portion of GPU memory. For instance, a read of size ~10 Kbases requires only ~18 MB of GPU memory while a read with 90 Kbases requires ~160MB memory. Hence, *very long reads* occupy a significant portion of GPU memory, limits the number of reads that could be processed in parallel. This reduces the amount of parallelism and the occupancy of the GPU is reduced.

Fortunately, *very long reads* being few (see the typical read length distribution under results), the CPU (core frequency faster than on GPU) could process those reads while GPU is processing the rest of the reads. In the above example, selecting a static threshold (eg: processing reads of length <30Kbases on GPU and rest on CPU) would give reasonable performance. However, selecting such a static threshold is not ideal due to variations in the read length distributions based on the dataset (see background). Thus, we use the product of *max-lf* and the average read length in the batch to determine the threshold dynamically, where *max-lf* is a user-parameter that defaults to 5.0. This threshold was empirically determined.

Now assume amongst the *very long reads* processed on the CPU, a few *ultra long reads* (eg: read >100 Kbases in a dataset where >99% of the reads are <100 Kbases). Such *ultra long reads* could cause a severe load imbalance between the CPU and the GPU. For instance, assume that there exists a read which is 1 Mbases in a given read batch. Despite the high core frequency, the CPU will take a few seconds to process such an *ultra long read*. The GPU meanwhile would process the whole batch in less than 1s (see results for

empirical evidence). Such *ultra long reads* being <1%, are skipped during the processing (while being written to a separate file) and are separately processed by the CPU at the end. In our implementation, the threshold for *ultra long reads* is a user defined parameter that defaults to 100 Kbases. There is an additional advantage of processing *ultra long reads* later. *Ultra long reads* usually require a significant amount of RAM (a few gigabytes) and may crash on limited memory systems. In the end, it is possible to process these reads with a limited amount of threads to reduce the peak memory consumption, particularly if the size of the RAM is limited.

S2.3.2 Over segmented reads

Once the *very long reads* and *ultra long reads* are processed as in Section S2.3.1, the performance impact due to the over-segmented events become prominent. While the majority of the reads have a number of events per base that is close to the average $\mu(= 2.5)$, a few reads can have a very large value. For instance, a few reads with a number of events per base being more than eight times the average $\mu(= 2.5)$ can violate the suitability of our partitioning of GPU memory as X and Y (X and Y are derived in equations 3 and 4). These over-segmented reads lead to the GPU arrays that are proportional to Y be full, while the arrays proportional to X are left under-utilised. For instance, arrays proportional to Y can become 100% while arrays proportional to X are only filled to <70%. Hence, over segmented reads lead to under-utilisation of GPU memory and results in limiting the number of reads which are processed in parallel. We process the over-segmented reads on the CPU based on a user specifiable threshold *max-epk* which defaults to 5.0.

On rare occasions, reads with >100 events per base were observed. Such severely over-segmented reads can be processed separately at the end or ignored totally as such rare reads amongst millions of other reads are unlikely to affect the final polishing result.

S2.3.3 Batch size

The selection of proper batch size (reads loaded to RAM from the disk at a time) is another important parameter that affects performance. If the batch size is too small compared to what the GPU memory can accommodate, the number of reads to be processed in parallel is limited, thus leads to in-adequate occupancy. Conversely, if the batch is too large to fit the GPU, CPU will have to process many surplus reads that could not be accommodated into the GPU. The batch size in our implementation is determined by two user specified parameters: K which is the maximum number of reads; and, B which is the maximum number of total bases. When reading from the disk to RAM, the true batch size (n-number of reads and b-number of total bases are capped by K and B) is determined by the first value (n or b) reaching the cap (K or B) first. Having such a limit B allows capping peak RAM due to adjacent *very long reads*. The suitable value for B is dependent on the available GPU memory, which can be estimated via equation 3 discussed in Section S2.2.

S2.3.4 Detection of performance anomalies

While we have empirically determined typical parameters/thresholds (associated with the above strategies), an unusual situation (for instance, a big gap between the CPU and GPU specifications or a data set that severely deviates from the heuristics we use) may cause performance anomalies. We employ the following method to detect a severe performance anomaly caused by such an unusual scenario.

We measure the quantities representing resource utilisation during run time, which are listed in Table S3. These quantities are measured per batch of reads loaded to the RAM at a time. We use those measured quantities to determine any severe performance issues and suggest suitable parameter adjustments to the user. The adjustable parameters (or thresholds) that can be tweaked to improve resource utilisation are defined in Table S4. Determination of performance issues and suggestions are done via two decision trees, one that corresponds to GPU memory usage (Fig. S8a) and another which corresponds to balancing the load between CPU and GPU (Fig. S8b).

Fig. S8a shows the decision tree that detects any imbalance in the proportions X and Y associated with GPU arrays allocation (X and Y derived in equations 3 and 4). The objective of this decision tree is to detect any GPU memory wastage and to increase the number of reads which the GPU gets to process in parallel.

As shown in Fig. S8a, if both X_{util} and Y_{util} (*rs* as a percentage of X and *es* as a percentage of Y in Algorithm S10) are more than 70%, the utilisation of GPU arrays is considered reasonable. Note that 70% is an empirically determined value that provides adequate performance. If X_{util} is reasonable (>70%) and Y_{util} is unreasonable (<70%), we inspect for any significant imbalance between X_{util} and Y_{util} (X_{util}-Y_{util}>30%). Such a significant gap suggests an under-utilisation, which should be remedied through the increase of *max-epk* (the threshold at which over-segmented reads are offloaded to the CPU) or reducing Y by decreasing *average-epk* (node S1 in Fig. S8a). In contrast, if Y_{util} is reasonable and the X_{util} is unreasonable, the strategy is the opposite, i.e, either decrease *max-epk* or increase *average-epk* (follow up to the node S2 in Fig. S8a).

quantity	description
t_{CPU}	processing time on CPU
t_{GPU}	processing time on GPU
X_{util}	utilisation percentage of the arrays proportional to X (*rs* as a percentage of X in Algorithm S10)
Y_{util}	utilisation percentage of the arrays proportional to Y (*es* as a percentage of Y in Algorithm S10)
N_{memout}	number of reads assigned to CPU due to GPU memory getting prematurely full (corresponds to line 11 of Algorithm S10)
N_{long}	number of *very long reads* assigned on to the CPU (corresponds to user parameter *max-lf*)
N_{events}	number of reads with too many events per read assigned onto the CPU (corresponds to user parameter *max-epk*)
n	the number of reads actually loaded to the RAM
b	the number of bases actually loaded to the RAM

Table S3: measured quantities

parameter	description
max-lf	reads with length $\leq$ *max-lf* $\times$ *average_read_length* are assigned to GPU and rest to CPU
avg-epk	average number of events per base used for allocating GPU arrays as discussed previously (μ)
max-epk	reads with events per base $\leq$ *max-epk* are assigned to GPU, rest to CPU
K	upper limit of the batch size with respect to the number of reads
B	upper limit of the batch size with respect to the number of bases
t	number of CPU threads
ultra-thresh	threshold to skip *ultra long reads*

Table S4: adjustable user parameters

If both X_{util} and Y_{util} are less than 70%, a likely cause is an inadequate batch size to fill the GPU memory. The actual batch size (n,b) is determined by both K and B as stated previously. As shown in Fig. S8a, we check which limit out of K and B was reached first. If both $n < K$ and $b < K$, the currently processed batch being the last batch in the dataset (end of input data reached) is the likely cause. Thus, no parameter tuning action is necessary. If B was reached first ($n < K$ and not $b < B$), B is the limitation and should be increased (S3 in Fig. S8a). If K was reached first (not $n < K$ and $b < B$), K should be increased (S4 in Fig. S8a).

Fig. S8b shows the decision tree for CPU-GPU workload balancing. For a particular batch, if the CPU takes significantly more time than the GPU, the decision tree first inspects whether the CPU is assigned with an excessive workload. An excessive workload on the CPU can be attributed by: an extensively over-sized batch size (in comparison to the available GPU memory), which results in a majority of the reads being assigned to the CPU (N_{memout}>10%); excessive number of *very long reads* assigned to the CPU (N_{long}>10%); and, excessive number of over-segmented reads events assigned to the CPU (N_{events}>10%). If N_{memout}>10%, K is reduced (node T1 in Fig. S8b); if N_{long}>10%, *max-lf* is increased (T2 in Fig. S8b); and, if N_{events}>10%, *max-epk* is increased (T3 in Fig. S8b).

If the cause for higher CPU time is not the aforementioned excessive workload, a likely cause is *ultra long reads*, where a single *ultra long reads* processed on the CPU taking more time than the time taken by GPU for the whole batch. In such an event, *ultra-thresh* threshold is reduced so that more *ultra long reads* are skipped. Another likely cause is that the program was executed with inadequate threads (if the CPU had more hardware threads than the program was launched), which is to be remedied by increasing the number of CPU threads. Another cause might be that the CPU is not sufficiently powerful to match with the GPU and thus no action can be taken (except upgrading the CPU). These actions are denoted by T4 in Fig. S8b.

The ideal case is when the CPU and GPU take similar times which requires no intervention.

Conversely, if the GPU takes significant time than the CPU, the likely causes are *very long reads* or over-segmented reads. In such an event, the thresholds *max-lf* and *max-epk* are decreased so that more *very long reads* and over-segmented reads are assigned to the CPU. Another likely cause is the *ultra long read* which can be remedied by increasing *ultra-thresh* threshold. Another cause might be an insufficiently powerful GPU (less compute cores or less memory) compared to the CPU and no action is taken (except to upgrade the GPU).

To reduce false positives due to incidental underutilisation, a suggestion is provided to the user, only if the same condition (the condition that led to the decision in the decision tree, S1 to S4 T1 in Fig. S8a and T1 to T4 in Fig. S8b) consecutively repeats more than a few times (eg: >3 times).

Note that the above mentioned strategy is to warn and suggest potential parameter adjustments in the event of drastic performance degradation, rather than

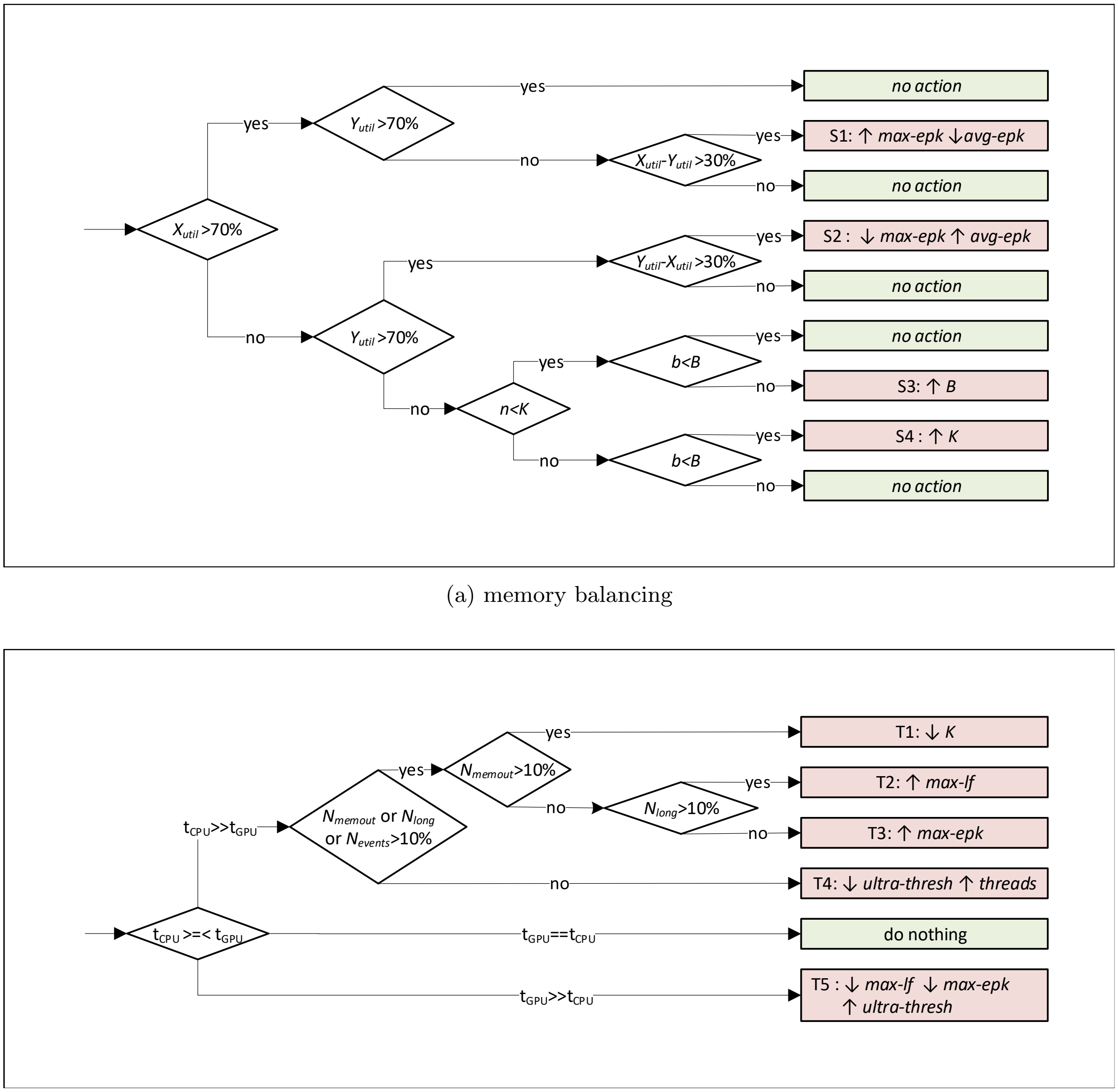

(a) memory balancing

(b) load balancing

Figure S8: Decision trees for resource optimisation

to obtain optimal performance or to determine the exact parameter values.

Algorithm S6 Adaptive Banded Event Alignment - *core-kernel*

```
function ALIGN_KERNEL_CORE(...)    ▷ ... refers to the arguments which are later explained in Section S2.2
    j ← thread index along x    ▷ the x subscript of a thread Fig. S5
    i ← thread index along y    ▷ the y subscript of a thread Fig. S5
    (events,score,trace,ll_idx,kcache) ← get_cuda_pointers(i,...)    ▷ get memory pointers of the arrays corresponding to read i (explained in Section S2.2
    n_bands ← n_events + read_len
    __shared__ c_score[W], p_score[W], pp_score[W]    ▷ allocate space in fast shared memory for scores of current, previous and 2nd previous bands
    __shared__ c_ll_idx, p_ll_idx, pp_ll_idx    ▷ allocate space in fast shared memory for indexes of lower left cells of current, previous and 2nd previous bands
    if (j<W) then    ▷ similar behaviour as in pre-kernel
        p_score[j],pp_score[j] ← score[1,j],score[0,j]    ▷ copy initialised b0 and b1 scores
        p_ll_idx,pp_ll_idx ← ll[1],ll[0]    ▷ copy initialised b0 and b1 indexes
        __syncthreads()    ▷ synchronise threads in the block
        for i ← 2 to n_bands do    ▷ similar to Algorithm S1
            if (j==0) then    ▷ only thread 0 process this
                dir ← suzuki_kasahara_rule(p_score)    ▷ similar to Algorithm S1
                if dir == right then
                    c_ll_idx ← move_band_to_right(p_ll_idx)    ▷ similar to Algorithm S1
                    ll[i] ← c_ll_idx    ▷ store to global memory
                else
                    c_ll_idx ← move_band_down(p_ll_idx)    ▷ similar to Algorithm S1
                    ll[i] ← c_ll_idx    ▷ store to global memory
                end if
            end if
            __syncthreads()    ▷ synchronise threads in the block
            min_j,max_j ← get_limits_in_band(c_ll_idx)    ▷ similar to Algorithm S1
            __syncthreads()    ▷ synchronise threads in the block
            if (j ≥ min_j AND j < max_j) then    ▷ fill the cells in band i in parallel
                s,d ← compute(p_score,pp_score,kcache,events,model)    ▷ see Algorithm S7
                c_score[j] ← s    ▷ store score to shared memory
                trace[i,j] ← d    ▷ store backtrack flag directly to global memory
            end if
            __syncthreads()    ▷ synchronise threads in the block
            score[i,j] ← c_score[j]    ▷ store the scores in global memory
            pp_score[j], p_score[j], c_score[j] ← p_score[j], c_score[j], −∞    ▷ update band scores for the next iteration
            if j==0 then
                pp_ll_idx, p_ll_idx ← p_ll_idx, c_ll_idx    ▷ update band indexes for the next iteration
            end if
            __syncthreads()    ▷ synchronise threads in the block
        end for
    end if
end function
```

Algorithm S7 Adaptive Banded Event Alignment - *core-kernel* - cell score computation

Constants:

$events\_per\_kmer = \frac{n\_events}{n\_kmers}$

$\epsilon = 1^{-10}$

$lp\_skip = \ln(\epsilon)$

$lp\_stay = \ln(1 - \frac{1}{events\_per\_kmer+1})$

$lp\_step = \ln(1.0 - e^{lp\_skip} - e^{lp\_stay})$

```
function COMPUTATION(score_prev,score_2ndprev,kcache,events)
    lp_emission ← log_probability_match(kcache,events)    ▷ see Algorithm S8
    up,diag,left ← get_scores(score_prev,score_2ndprev)    ▷ see red arrows in Fig. S3f
    score_d ← diag + lp_step + lp_emission
    score_u ← up + lp_stay + lp_emission
    score_l ← left + lp_skip
    s ← max(score_d,score_u,score_l)
    d ← direction from which the max score came
end function
```

Note: Changes to Algorithm S2 are highlighted in blue

Algorithm S8 Adaptive Banded Event Alignment - *core-kernel* - log probability computation.

```
function LOG_PROBABILITY_MATCH(kcache,events)
    event ← get_event(events)    ▷ see red arrow in Fig. S3f
    x ← event.mean
    model_kmer ← get_entry_from_kcache(kcache)
    μ ← model_kmer.mean
    σ ← model_kmer.stdv
    z ← (x−μ)/σ
    lp_emission ← ln(1/√(2π)) − ln(σ) − 0.5z²
end function
```

Note: Changes to Algorithm S3 are highlighted in blue

Algorithm S9 Memory allocation—data structure serialisation

```
for batch of n reads do
    ...    ▷ CPU processing steps before the ABEA eg: event detection
    rs, es ← 0, 0    ▷ cumulative sum of read lengths and no of events
    for each read i do
        p[i], q[i] ← rs, es    ▷ save current read and event offsets
        rs ← rs + r[i]; es ← es + e[i]
    end for
    serialise_ram_arays(p, q, ...)    ▷ flatten multi dimensional arrays in RAM to 1D arrays
    allocate_gpu_arrays(rs,es,...)    ▷ GPU arrays REF, KCACHE, EVENTS, etc.
    memcpy_ram_to_gpu(...)    ▷ copy inputs of the ABEA to the GPU memory
    gpu_alignment(p, q...)    ▷ Perform ABEA on the GPU
    memcpy_gpu_to_ram(...)    ▷ copy alignment result back to the RAM
    deserialise(p, q, ....)    ▷ convert 1D result array to multi dimensional array
    free_gpu_arrays()    ▷ free GPU arrays REF, KCACHE, EVENTS, etc.
    ...    ▷ CPU processing steps after ABEA eg: HMM
end for
```

Algorithm S10 heuristic memory allocation scheme

allocate_gpu_arrays(X,Y) ▷ pre-allocate GPU arrays REF, KCACHE, EVENTS, etc.
for batch of n reads **do**
  ... ▷ CPU processing steps before the ABEA eg: event detection
  $rs, es \leftarrow 0, 0$ ▷ cumulative sum of read lengths and no of events
  for each read i **do**
    if $(rs + r[i] \leq X$ **and** $es + e[i] \leq Y)$ **then** ▷ check if GPU arrays have adequate free space
      $p[i], q[i] \leftarrow rs, es$ ▷ save current read and event offsets
      $rs \leftarrow rs + r[i]; es \leftarrow es + e[i]$
      assign_to_gpu(i) ▷ GPU arrays have space, thus assign read to the GPU
    else
      assign_to_cpu(i) ▷ a GPU arrays is already full, thus assign the read to the CPU
    end if
  end for
  $serialise\_ram\_arays(p, q, ...)$ ▷ flatten multi dimensional arrays in RAM to 1D arrays
  $memcpy\_ram\_to\_gpu(...)$ ▷ copy inputs of the ABEA to the GPU memory
  $gpu\_alignment(p, q...)$ ▷ Perform ABEA on the GPU
  process_rest_on_cpu() ▷ execute on the CPU in parallel to the GPU kernels
  $memcpy\_gpu\_to\_ram(...)$ ▷ copy alignment result back to the RAM
  $deserialise(p, q, ...)$ ▷ convert 1D result array to multi dimensional array
  ... ▷ CPU processing steps after ABEA eg: HMM
end for
free_gpu_arrays() ▷ free GPU arrays REF, KCACHE, EVENTS, etc.

Note: Changes to Algorithm S9 are highlighted in blue

S3 Extended Results

Experimental setup is given in Section S3.1. In Section S3.2, we present experimental evidence that justifies the selection of steps presented in Section S2.

S3.1 Experimental setup

We re-engineered the *Nanopolish* methylation calling tool (existing methylation detection tool discussed in Section S1) to: one, load a batch of n reads from disk to RAM at a time, instead of on-demand loading; two, synchronise CPU threads prior to GPU kernel invocation (*Nanopolish* assigns a thread dynamically to a particular thread, thus each read follows its own code path); and three, optimise the CPU implementation which otherwise would result in an apparent un-fair speedup (when the optimised GPU version is compared to an un-optimised CPU version).

We used publicly available NA12878 (human genome) Nanopore WGS Consortium sequencing data [11] for the experiments. The datasets used for the experiments, their statistics (number of reads, total bases, mean read length and maximum read length) and their source are listed in Table S5.

D_{small} dataset was used for experiments under Sections S3.2.2, S3.2.1 and D_{rapid} for Section S3.2.3.

To obtain the results for Section S3.2.3, first, we grouped the reads in dataset D_{rapid} based on their read lengths. We grouped the read into 10 Kbases bins (i.e., 0K-10K,10K-20K...90K-100K). Reads with >100 Kbases were grouped into larger bins (100K bin sizes; 100K-200K, 200K-300K and 200K-300K) as the read count is very little in the range that certain 10K bins would contain no reads at all. Then, we ran *f5c* with only CPU and *f5c* with GPU acceleration on each group of the reads separately. Then, we computed the speedup of ABEA for each group of reads: the kernel only speedup (*GPU kernel time / time on CPU*); and, the speedup with overheads (overheads such as memory copy, data structure serialisation). This experiment was performed on the system *lapH*.

For Sections S3.2 time measurements were obtained by inserting *gettimeofday* function invocations directly into the C source code.

S3.2 Effect of individual optimisations

S3.2.1 Compute optimisations

Fig. S8c shows the time consumed by the three GPU kernels after applying the compute optimisation techniques discussed in Section S2.1. Time taken by each of the three GPU kernels (*pre-kernel*, *core-kernel* and *post-kernel*) is plotted for each different GPU. It is observed that the *core-kernel*, which computes the dynamic programming table (compute-intensive portion), still consumes the majority of the GPU compute time. The *pre-kernel* which performs data structure initialisation consumes much lesser time and shows that there is no need to further parallelise the loop in Algorithm S5 (explained in Section S2.1). Despite the lack of fine-grained parallelism in *post-kernel* (which performs backtracking), the elapsed time is still considerably lesser than the *core-kernel*. Thus, any future optimisations should still mainly focus on the *core-kernel*, followed by the *post-kernel*.

The efficacy of our compute optimisations on the compute intensive *core-kernel* can be elaborated using the reported statistics from the NVIDIA profiler (instruction level profiling—PC sampling in NVIDIA visual profiler [20]). The profiler reports the percentage distribution of reasons that caused the thread warps to stall, based on the number of clock cycles. The percentage of the number of clock cycles that a warp was stalled due to a memory dependency (waiting for a previous memory accesses to complete), improved from 59.10% to 44.81% after the use of GPU shared memory. After exploiting the *kcache* for improving memory coalescing, this percentage further improved to 28.62%.

S3.2.2 Memory optimisations

As stated in Section S2.2.1, the data array serialisation technique eliminated all memory allocations inside GPU kernels (*malloc*); still, required memory allocations per each batch of reads (*cudaMalloc*). The overheads due to these *cudaMalloc* calls are plotted in Fig. S8d along with the time for kernel execution and data transfer to/from the GPU (using *cudaMemcpy*). Observe that on certain GPUs (Jetson TX2, GeForce 940M and Tesla K40), the overheads due to *cudaMalloc* operations are significant in comparison to the compute kernels (even higher than the compute kernels in Jetson TX2). Such significant overheads justify why we proposed a heuristic based memory pre-allocation technique (Section S2.2.2) which completely eliminates this overhead.

Interestingly, Tesla K40 and Geforce 940M which incurred high *cudaMalloc* overheads are of relatively older GPU architectures in comparison to GeForce 1050 and Tesla V100, where the overheads were minimal. This is probably due to hardware supported memory allocation in latest GPU architectures. However, the aforementioned observation seems to be valid only for GeForce GPUs (targeted for gaming on PC/laptops) and Tesla GPUs (targeted for high performance computing). On Tegra GPUs (SoC targeted for embedded devices) the overhead seems to be significant in spite of the latest architectures (Jetson TX2 is the same Pascal architecture as GeForce 1050). We additionally tested on a Jetson AGX Xavier (the most

Table S5: Information of the datasets

Dataset	Number of reads	Number of bases (Gbases)	Mean read length (Kbases)	Max read length (Kbases)	Source / SRA accession
D_{small}	19275	0.15	7.7	196	[19]
$D_{ligation}$	451020	3.62	8.0	1500	ERR2184733
D_{rapid}	270189	2.73	10.0	386	ERR2184734

Table S6: Different systems used for experiments

System Name	Info	CPU	CPU cores / threads	RAM (GB)	GPU	GPU mem (GB)	GPU arch
SoC	NVIDIA Jetson TX2 embedded module	ARMv8 Cortex-A57 + NVIDIA Denver2	6 / 6	8	Tegra	shared with RAM	Pascal / 6.2
lapL	Acer F5-573G laptop	i7-7500U	2/4	8	Geforce 940M	4	Maxwell / 5.0
lapH	Dell XPS 15 laptop	i7-8750H	6/12	16	Geforce 1050 Ti	4	Pascal / 6.1
ws	HP Z640 workstation	Xeon E5-1630	4/8	32	Tesla K40	12	Kepler / 3.5
HPC	Dell PowerEdge C4140	Xeon Silver 4114	20/40	376	Tesla V100	16	Volta / 7.0

recent Tegra GPU based SoC — Volta architecture) and *cudaMalloc* was yet expensive (40s on GPU kernels and 44s on *cudaMalloc*, not shown in figure). Thus, our memory pre-allocation strategy (in Section S2.2.2) which totally eliminates this *cudaMalloc* overhead is specifically beneficial for GPU on SoCs.

S3.2.3 Heterogeneous processing

We stated in Section S2.3 that *very long reads* if processed on the GPU, limits the GPU occupancy. Fig. S8e provides experimental evidence and shows the need to process *very long reads* on CPU (explained in Section S2.3). Fig. S8e plots the variation of the speedup (GPU compared to CPU for ABEA) as the read length varies. The x-axis labels the range of the read length for which the speedup was computed (explained in the experimental setup). For instance, 0-10 on the x-axis refers to the group of reads with read length 0-10Kbases. Note that in Fig. S8e the bins are 100K wide from 100K-200K on-wards, due to less number of reads of those lengths (explained in the experimental setup). The speedup of *computations* (GPU kernel time / CPU time) and the speedup including *overheads* (GPU kernel time + overheads such as memory copy, data structure serialisation) are plotted in Fig. S8e. Speedup of more than 4X was observed for smaller read lengths (0-10K). speedup drops with increasing read-length and is less than 3X from 50K-60K. The longer the reads are, the lesser number of reads can be processed in the GPU in parallel (reduced occupancy), thus the reduced speedup. Hence, *very long reads* that significantly affects the performance should be performed on the CPU while the GPU is processing the rest.

Fig. S8f shows the need for processing *ultra long reads* separately (explained in Section S2.3). The x-axis in the figure is the read-length (similar to Fig. S8e). The blue bars (with reference to the right y-axis) denote the average time consumed by the GPU to process a batch of reads (1.5 Mbases), for each group of read lengths from 0 bases to 50Kbases. The orange bars (with reference to the right y-axis) denote the average time consumed by the CPU (1 thread) to process a single read in the particular group of reads. The read length distribution (left y-axis) is shown shaded in green colour to depict the abundance of reads in each read length. Observe that CPU takes >1.6s for a single read of 300K-400K length while the GPU completes a whole 40K-50K batch in <0.4s. Thus, the GPU would idle for >1.2s until the CPU completes processing. Hence, such *ultra long reads* (eg : >100 Kbases) must be skipped and processed separately at the end. Note that such *ultra long reads* are very few (green coloured read length distribution in Fig. S8f).

S4 Miscellaneous

S4.1 Why Nanopolish had to be re-engineered?

There are three reasons why *Nanopolish* had to be completely re-engineered into *f5c* for a successful GPU implementation.

- *Nanopolish* performs on-demand loading of signal data from file (a CPU thread assigned to the particular read invokes a file access just prior to signal alignment). However, transferring read by read to

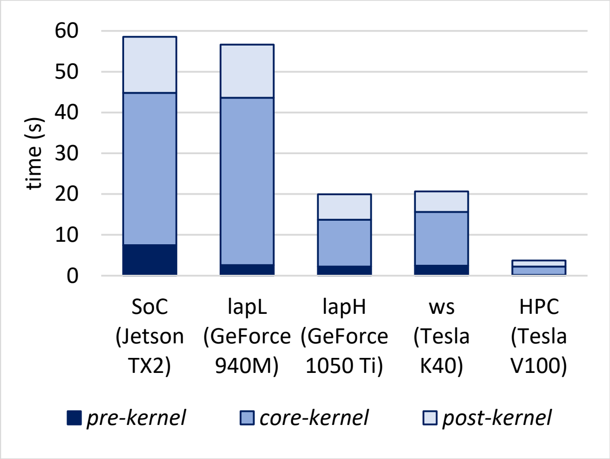

(c) Distribution of GPU kernel execution time

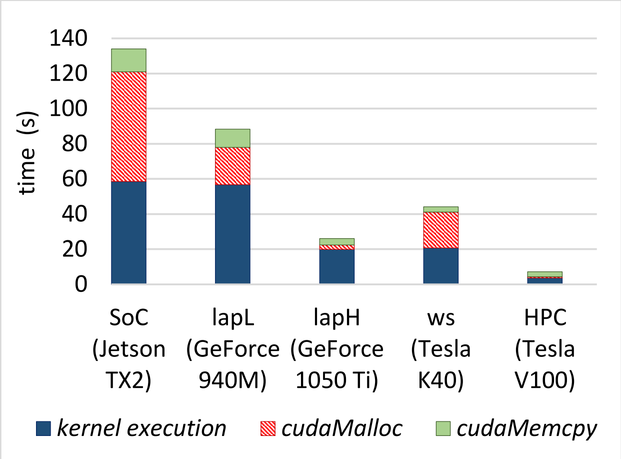

(d) Time for GPU kernels compared *cudaMalloc*

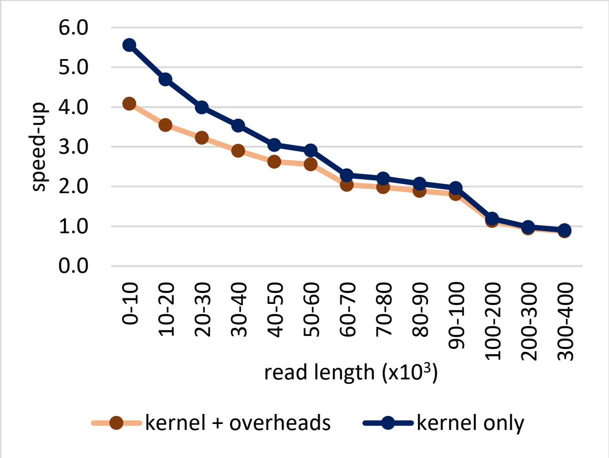

(e) Effect of the read length on the speedup

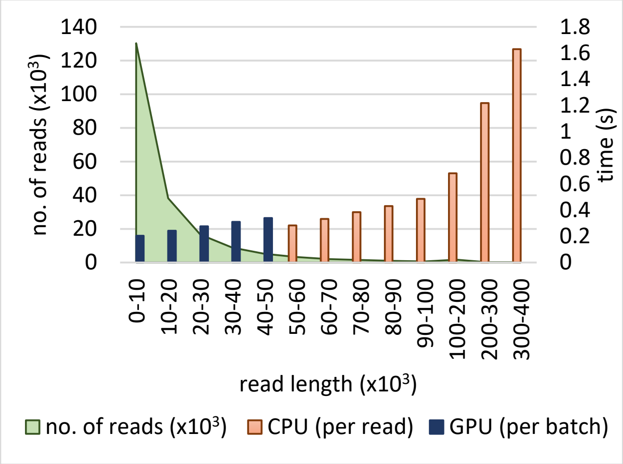

(f) need for load-balancing based on read lengths

Figure S9: Effect of individual optimisations

the GPU will incur a massive penalty and thus a batch of reads have to be transferred at once. Thus, we had to re-write the Nanopolish processing framework in such a way that loading and processing of a batch are performed batch wise. In *f5c*, we read a batch of data to the RAM and then bulk transfer to GPU memory, a batch of n reads at a time.

- Nanopolish thread model un-suitable for GPU acceleration—a thread is dynamically assigned to a read using openMP, thus each read has its own code path. However, offloading a batch of reads to the GPU for signal alignment requires code paths of all the reads in the batch to have converged before the GPU kernel is invoked. In addition, accurately measuring time, benchmarking and profiling of individual algorithmic components is hindered by such divergent code paths. *pthread* based approach that interleaves input reading, processing and output.
- Nanopolish is not optimised for efficient resource utilisation (eg: marginal performance improvement beyond 16 threads on servers and heavyweight for embedded systems due to spurious *malloc* calls). A comparison of such a version with the GPU would result in an apparent high speedup, which is unfair.

S4.2 Additional advantages of *f5c* over *Nanopolish*

In addition to the GPU acceleration of ABEA, *f5c* has many additional advantages over original *Nanopolish*.

- I/O and processing are interleaved in *f5c*: the I/O latency is considerably minimised.

- Our CPU version alone is around 1.5X-2X faster than the *Nanopolish* call methylation implementation and is very lightweight - suitable for embedded systems due to the careful use of data structures and algorithms.
- *f5c* is capable of detecting load balance problems between CPU and GPU, and report user with suggestion for appropriate parameters.
- *f5c* works with package manager's system wide installations of HDF5 (no need of thread-safe build of HDF5), hence no need locally compile HDF5.
- Dependency hell has been minimised for both CPU and GPU versions. Compatible with g++ 4.8 or higher, and CUDA toolkit 6.5 or higher.
- *f5c* has suggestive error message for troubleshooting, especially the issues with respect to GPU.
- *Pthread* based thread framework written in C that interleaves I/O with processing is very lightweight and can be a starting point for future Nanopore tools.
- *f5c* allows benchmarking section by section to identify the bottlenecks in performance.
- *f5c* framework is suitable for the acceleration of core kernels through other methods such as FPGA.

Author details

[1]School of Computer Science and Engineering, University of New South Wales, Sydney, Australia. [2]Kinghorn Centre for Clinical Genomics, Garvan Institute of Medical Research, Sydney, Australia. [3]Department of Computer Engineering, University of Peradeniya, Peradeniya, Sri Lanka. [4]St-Vincent's Clinical School, Faculty of Medicine, University of New South Wales, Sydney, Australia. [5]Ontario Institute for Cancer Research, Toronto, Canada. [6]Department of Computer Science, University of Toronto, Toronto, Canada.

References

1. Liyanage, V., Jarmasz, J., Murugeshan, N., Del Bigio, M., Rastegar, M., Davie, J.: Dna modifications: function and applications in normal and disease states. Biology **3**(4), 670–723 (2014)
2. Lewandowska, J., Bartoszek, A.: Dna methylation in cancer development, diagnosis and therapy—multiple opportunities for genotoxic agents to act as methylome disruptors or remediators. Mutagenesis **26**(4), 475–487 (2011)
3. Fraser, M., Sabelnykova, V.Y., Yamaguchi, T.N., Heisler, L.E., Livingstone, J., Huang, V., Shiah, Y.-J., Yousif, F., Lin, X., Masella, A.P., *et al.*: Genomic hallmarks of localized, non-indolent prostate cancer. Nature **541**(7637), 359 (2017)
4. Saxonov, S., Berg, P., Brutlag, D.L.: A genome-wide analysis of cpg dinucleotides in the human genome distinguishes two distinct classes of promoters. Proceedings of the National Academy of Sciences **103**(5), 1412–1417 (2006)
5. Bird, A.: Dna methylation patterns and epigenetic memory. Genes & development **16**(1), 6–21 (2002)
6. Gonzalo, S.: Epigenetic alterations in aging. Journal of applied physiology **109**(2), 586–597 (2010)
7. Lu, H., Giordano, F., Ning, Z.: Oxford nanopore minion sequencing and genome assembly. Genomics, proteomics & bioinformatics **14**(5), 265–279 (2016)
8. Rang, F.J., Kloosterman, W.P., de Ridder, J.: From squiggle to basepair: computational approaches for improving nanopore sequencing read accuracy. Genome biology **19**(1), 90 (2018)
9. Wick, R.R., Judd, L.M., Holt, K.E.: Performance of neural network basecalling tools for oxford nanopore sequencing. bioRxiv, 543439 (2019)
10. Li, H.: Minimap2: pairwise alignment for nucleotide sequences. Bioinformatics, 191 (2018). doi:10.1093/bioinformatics/bty191
11. Jain, M., Koren, S., Miga, K.H., Quick, J., Rand, A.C., Sasani, T.A., Tyson, J.R., Beggs, A.D., Dilthey, A.T., Fiddes, I.T., *et al.*: Nanopore sequencing and assembly of a human genome with ultra-long reads. Nature biotechnology **36**(4), 338 (2018)
12. Loman, N.J., Quick, J., Simpson, J.T.: A complete bacterial genome assembled de novo using only nanopore sequencing data. Nature methods **12**(8), 733 (2015)
13. Simpson, J.T., Workman, R.E., Zuzarte, P., David, M., Dursi, L., Timp, W.: Detecting dna cytosine methylation using nanopore sequencing. Nature methods **14**(4), 407 (2017)
14. Durbin, R., Eddy, S.R., Krogh, A., Mitchison, G.: Biological Sequence Analysis: Probabilistic Models of Proteins and Nucleic Acids. Cambridge university press, ??? (1998)
15. Suzuki, H., Kasahara, M.: Introducing difference recurrence relations for faster semi-global alignment of long sequences. BMC bioinformatics **19**(1), 45 (2018)
16. David, M., Dursi, L.J., Yao, D., Boutros, P.C., Simpson, J.T.: Nanocall: an open source basecaller for oxford nanopore sequencing data. Bioinformatics **33**(1), 49–55 (2016)
17. NVIDIA: CUDA C Programming Guide. (2018). PG-02829-001_v10.0
18. NVIDIA: CUDA C Best Practices Guide. (2018). DG-05603-001_v10.0
19. Simpson, J.: Stats and Analysis (2017). https://nanopolish.readthedocs.io/en/latest/quickstart_call_methylation.html
20. NVIDIA: PROFILER USER'S GUIDE. (2019). DU-05982-001_v10.1
